# RNAi-mediated silencing of MLL-AF9 reveals leukemia-associated downstream targets and processes

**DOI:** 10.1186/1476-4598-13-27

**Published:** 2014-02-11

**Authors:** Katrin K Fleischmann, Philipp Pagel, Irene Schmid, Adelbert A Roscher

**Affiliations:** 1 Children’s Research Center, Division of Pediatric Hematology and Oncology, Dr. von Hauner Children’s Hospital, Ludwig-Maximilians-Universität München, Lindwurmstrasse 2a, München 80337, Germany; 2 Lehrstuhl für Genomorientierte Bioinformatik, Technische Universität München, Maximus-von-Imhof-Forum 3, Freising D-85354, Germany; 3 Children’s Research Center, Dr. von Hauner Children’s Hospital, Ludwig-Maximilians-Universität München, Lindwurmstrasse 2a, München 80337, Germany

**Keywords:** Acute myeloid leukemia, MLL-AF9, Fusion gene, siRNA, Gene expression, Therapeutic targets, Molecular processes, SCH39166, Ecopipam

## Abstract

**Background:**

The translocation t(9;11)(p22;q23) leading to the leukemogenic fusion gene *MLL-AF9* is a frequent translocation in infant acute myeloid leukemia (AML). This study aimed to identify genes and molecular processes downstream of MLL-AF9 (alias MLL-MLLT3) which could assist to develop new targeted therapies for such leukemia with unfavorable prognosis.

**Methods:**

In the AML cell line THP1 which harbors this t(9;11) translocation, endogenous *MLL-AF9* was silenced via siRNA while ensuring specificity of the knockdown and its efficiency on functional protein level.

**Results:**

The differential gene expression profile was validated for leukemia-association by gene set enrichment analysis of published gene sets from patient studies and *MLL-AF9* overexpression studies and revealed 425 differentially expressed genes. Gene ontology analysis was consistent with a more differentiated state of MLL-AF9 depleted cells, with involvement of a wide range of downstream transcriptional regulators and with defined functional processes such as ribosomal biogenesis, chaperone binding, calcium homeostasis and estrogen response. We prioritized 41 gene products as candidate targets including several novel and potentially druggable effectors of MLL-AF9 (AHR, ATP2B2, DRD5, HIPK2, PARP8, ROR2 and TAS1R3). Applying the antagonist SCH39166 against the dopamine receptor DRD5 resulted in reduced leukemic cell characteristics of THP1 cells.

**Conclusion:**

Besides potential new therapeutic targets, the described transcription profile shaped by MLL-AF9 provides an information source into the molecular processes altered in *MLL* aberrant leukemia.

## Background

The *MLL* gene has been found translocated to over 50 different partner genes in acute leukemia. Certain partner genes are associated with distinct leukemia subtypes, e.g. *MLL-AF4* with pro B acute lymphoblastic leukemia (ALL) and *MLL-AF9*, -*AF6* and -*AF10* with acute myeloid leukemia (AML) of M4 and M5 subtypes (French–American–British classification) [1]. *MLL-AF9* (alias *MLL-MLLT3*) results from the translocation t(9;11)(p22;q23) and is sufficient to initiate acute leukemia in murine models with potential secondary mutations being rapidly acquired [2,3]. MLL and AF9 wildtype proteins play essential roles in embryogenesis and hematopoiesis [4-6] and are parts of protein complexes leading to transcriptional initiation (MLL) and elongation (AF9) of target genes [7,8]. The fusion protein MLL-AF9 is believed to combine these properties, leading to increased activation of target genes via transcriptional initiation and elongation. *MLL-AF9* is the most frequent fusion gene in infant AML and especially associated with monoblastic AML (M5) [9,10]. New targeted therapies are needed for this type of leukemia with poor prognosis.

Novel therapeutic strategies which aim to intervene with DNA binding of MLL directly or with the assembly of MLL fusion protein with elongation complexes have been discussed [7]. However, drug targeting of protein-protein or protein-DNA interactions is difficult as can be seen by the fact that they are not frequently targeted by approved drugs [11]. An additional concern is that the wildtype functions of the involved proteins would be abrogated as well, leading to toxicity [7].

For these reasons, we specifically explored downstream effects of MLL-AF9 in order to identify new alternative drug targets for *MLL-AF9* positive AML. Some previous studies have analyzed targets of MLL-AF9 in *in vivo* mouse models [3,12,13] or through lentiviral *MLL-AF9* transduction [14]. *MLL-AF9* expression and the associated leukemogenic potential is significantly higher after retroviral transduction as compared to Mll-AF9 knockin and impacts biologic properties like myeloid colony formation and long term self-renewal capacity [3]. We thus altered the endogenous level of MLL-AF9 through specific and efficient siRNA knockdown in the human monoblastic cell line THP1 and studied the downstream effects. The comprehensive gene expression profile after MLL-AF9 depletion suggested several cellular processes and 41 genes as likely mediators of MLL-AF9 leukemogenic effects. Among those, seven gene products were selected as candidate drug targets. Functional relevance of one of these, the dopamine receptor DRD5, was confirmed as an antagonist resulted in reduced leukemic cell characteristics of THP1 cells.

## Results

### Specific siRNA knockdown of *MLL-AF9*

**Figure 1:**
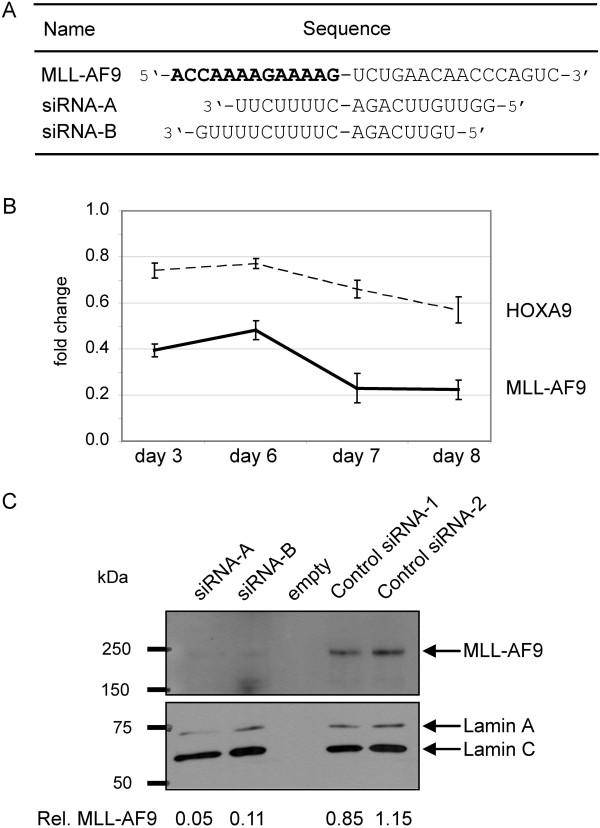
**SiRNA knockdown of MLL-AF9 in THP1 cells. (A)** Alignments of siRNAs to MLL-AF9 mRNA. Bold sequence represents the MLL part of the fusion transcript. **(B)** Confirmation of MLL-AF9 knockdown via RT-qPCR. Mean relative *MLL-AF9* and *HOXA9* transcript levels of knockdown (siRNA-A, siRNA-B) as compared to control treatments (control siRNA-1, -2) during the time course of experiments. Graph represents data from five independent experiments. Bars indicate standard deviation. **(C)** Immunoblot of MLL-AF9 from total protein extracted from day 8 experimental samples. Normalized MLL-AF9 levels are indicated at the bottom.

To specifically target *MLL-AF9* without disturbing *MLL* and *AF9* wildtype expression levels, siRNAs were designed to target the THP1 specific fusion point of *MLL-AF9* transcript (Figure 1A). Off-target effects were controlled by utilizing two different *MLL-AF9* specific as well as two different non-targeting control siRNAs. An experimental setup with prolonged knockdown of *MLL-AF9* (over 8 days) was chosen, because (1) the half-life of MLL-AF9 protein is unknown and (2) MLL-AF9 is believed to lead to changes in the expression of target genes via epigenetic mechanisms [7,8] whose reversal may take a long time [15] thus leading to a delayed effect on transcriptional level.

Mean transfection efficiency of THP1 cells was 93% and mean cell viability after transfection was likewise 93%. Knockdown of *MLL-AF9* reduced the transcript levels on day 8 of experiments to 22.3 ± 6% residual expression (Figure 1B). *MLL* and *AF9* wildtype transcript levels were not significantly altered.

To ensure an effective *MLL-AF9* knockdown on functional protein level, we quantified *HOXA9*: Transcription of *HOXA9* is raised by MLL-AF9 through direct interaction between MLL-AF9 protein complex and the *HOXA9* promoter [16,17]. *HOXA9* mRNA was reduced to 56.9 ± 8% residual expression on day 8 of *MLL-AF9* knockdown (Figure 1B). Additionally, immunoblotting confirmed an efficient reduction of MLL-AF9 on protein level (Figure 1C).

### Differentially expressed genes after *MLL-AF9* knockdown

The prolonged knockdown of *MLL-AF9* in THP1 cells yielded 571 probes representing transcripts of 425 genes as differentially expressed between knockdown and control treatments (Additional file 1, includes all accession numbers). Gene expression profiling data have been deposited in NCBI’s Gene Expression Omnibus (GEO) [18] and are available under the accession number GSE36592.

A number of previously defined criteria [19] supported the high quality of our gene expression data. These included the presence of important marker genes as expected for the study (i.e. probes detecting *MLL-AF9* and transcripts of the *HOXA* cluster), a reasonable number of differentially expressed genes which were enriched in certain biological processes as well as a significant correlation between microarray and RT-qPCR data of independent experiments (*p* 0.004, Spearman’s *Rho* 0.72).

**Figure 2:**
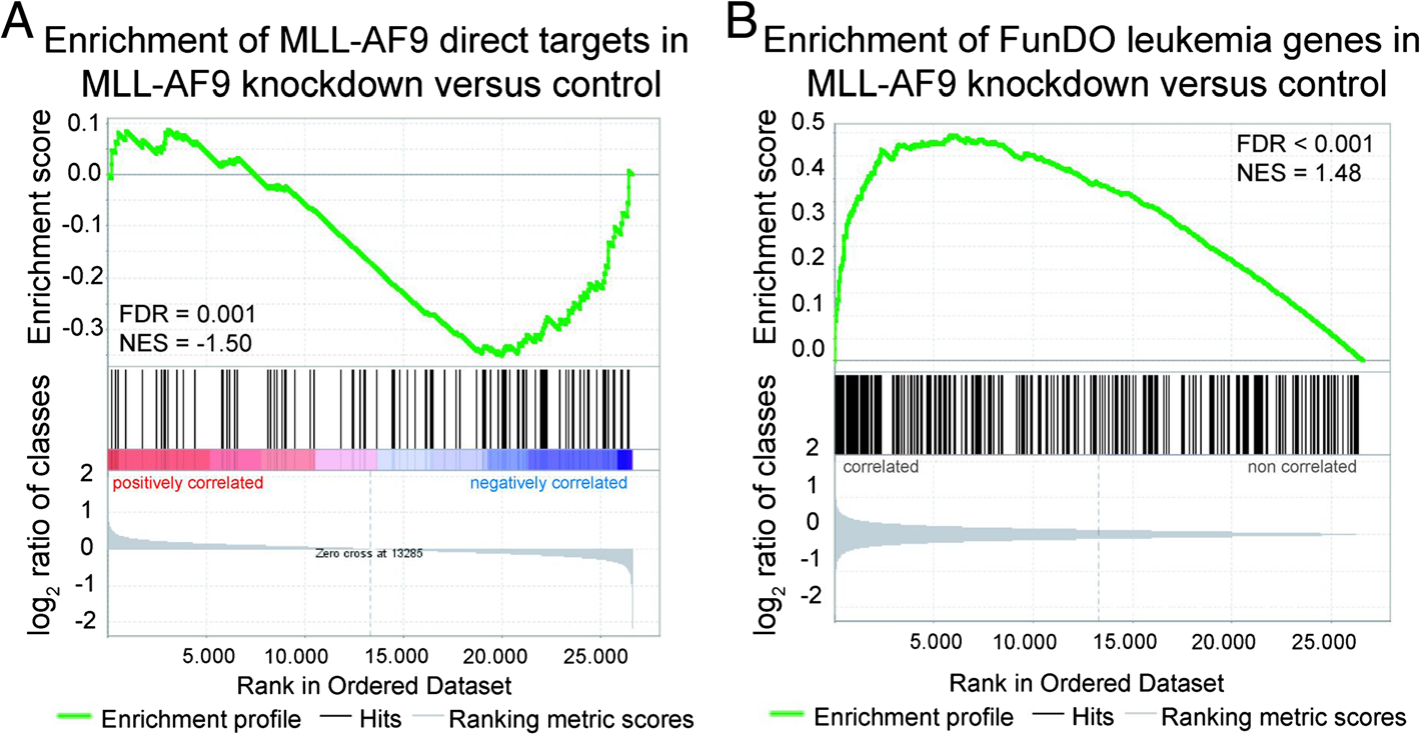
**Gene set enrichment plots for our gene expression profile after knockdown of *MLL-AF9 *in THP1 cells. (A)** Gene set enrichment plot of direct MLL-AF9 targets previously detected by Bernt et al. [13] in a mouse model. **(B)** Gene set enrichment plot of the leukemia gene set defined by FunDO. Because MLL-AF9 acts as a transcriptional activator, enrichment of direct targets was analyzed for the set of up- or down-regulated genes **(A)**, while the gene set altered in leukemia was analyzed for deregulated genes in general **(B)**. FDR, false discovery rate q-value; NES, normalized enrichment score.

We further validated our entire expression data set for concordance with known gene regulatory effects of MLL-AF9 via gene set enrichment analysis (GSEA). We detected significant enrichments of direct MLL-AF9 targets identified in a mouse model [13] (Figure 2A) and of genes downstream of MLL-AF9 identified in transduced primary human cells [14] (Additional file 2). In the latter, we observed a stronger enrichment of gene sets generated from human neonatal CD34+ cells as compared to adult CD34+ cells.

### Genes differentially expressed after *MLL-AF9* knockdown are associated with leukemia

The functional disease ontology database (FunDO) contains curated preexisting evidence on genes associated with disease entities. Applying FunDO analysis on our set of 425 differentially expressed genes, *Leukemia* was the most significant disease term associated with MLL-AF9 depletion (fold enrichment (FE) = 7.7; Bonferroni corrected *p*-value = 9.7 × 10^-11^). GSEA, which takes into account the entire gene expression profile without employing a significance cutoff value, confirmed the leukemia gene set predefined by FunDO as being significantly enriched (Figure 2B).

We further asked if our differential gene expression profile after *MLL-AF9* knockdown in THP1 cells is in agreement with preexisting knowledge from *in vivo* studies. Using GSEA, we found accordance (FDR *q*-value < 0.05) to *MLL*-aberrant and myeloid leukemia patient studies [1,20-23] (Table 1 and Additional file 3: Figure S1).

**Table 1:** **Gene set enrichment analysis of leukemia patient studies for our *MLL-AF9 *** **knockdown gene expression dataset**

**Patient study gene sets**	**Reference**	**FDR q-value**	**NES**
Top-100 gene set of *MLL* AML	Kohlmann et al. [1]	0.008	1.48
Top-100 gene set of *MLL* leukemia irrespective of lineage	Kohlmann et al. [1]	0.020	1.40
Top-100 probe set of pediatric *MLL* AML	Ross et al. [20]	0.022	1.38
Downregulated in *MLL* AML	Rozovskaia et al. [21]	0.005	1.69
Gene cluster 1 and 16 of 11q23 abnormal AML	Valk et al. [22]	0.030	1.34
CML-BP versus CML-CP	Zheng et al. [23]	<0.001	1.96
Downregulated in CML-BP versus CML-CP	Zheng et al. [23]	<0.001	2.08

Corresponding gene set enrichment plots are displayed in Additional file 3: Figure S1. *MLL* AML, *MLL* aberrant acute myeloid leukemia; CML-BP, chronic myeloid leukemia blastic phase; CML-CP, chronic myeloid leukemia chronic phase; FDR, false discovery rate; NES, normalized enrichment score.

### MLL-AF9 regulated genes are involved in defined cellular processes

**Figure 3:**
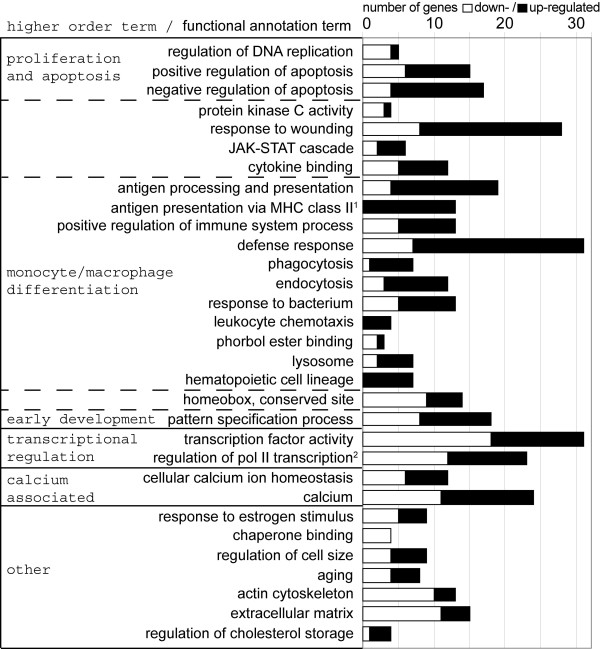
***MLL-AF9 *knockdown associated enriched functional gene annotations identified via DAVID.** Enriched annotation terms (fold enrichment (FE) ≥ 1.5 and *p* < 0.1) were manually assorted to six higher-order terms according to the major role of the process in the biological setting under investigation. Annotation terms assigned to two higher-order terms are indicated by dotted division lines between these two. Annotation terms which could not be assigned to one of the five specific higher-order terms are depicted under the term “other”. Some annotations were abbreviated as indicated by superscript numbers: ^1^Antigen processing and presentation of peptide or polysaccharide antigen via MHC class II, ^2^Regulation of transcription from RNA polymerase II promoter. Columns show proportion of down- (white) and up-regulated (black) genes after *MLL-AF9* knockdown in THP1 cells. All differentially expressed genes contained in each functional annotation term are given in Additional file 4 together with the term identifier, FE and *p* value of the terms.

Functional bioinformatic analysis via DAVID resulted in 31 enriched gene ontology annotation terms with potential biological relevance (Figure 3, Additional file 4). These terms were selected out of 312 significantly enriched annotations (Additional file 4) by omitting related and redundant terms and by evaluating their biological relevance as recommended [19]. To structure the results, the enriched annotation terms were manually assorted to functional higher-order terms according to their major role in the biological setting under investigation (Figure 3 and details in Additional file 3: Table S1).

The higher-order term *proliferation and apoptosis* contained 7 annotation-terms and suggested influence of MLL-AF9 on cell replication (*regulation of DNA replication*), proliferation (*JAK-STAT cascade*, *response to wounding, cytokine binding*) and apoptosis (*regulation of apoptosis*, *protein kinase C activity*). Several genes involved in replication or anti-apoptotic processes were down-regulated (*CALR*, *NPM2*, *POT1*, *STRA8* and *MEF2C, SOCS2*, *SOX4* respectively) while several genes encoding pro-apoptotic regulators were up-regulated (*CEBPB*, *DUSP1*, *HIPK2*, *LCK*, *NOTCH2*, *PRKCE*, *TGFBR1*, *TRIO*, *VDR*).

The higher-order term *monocyte/macrophage differentiation* included 16 annotation terms that encompassed fundamental functions of monocytes and macrophages like *antigen processing and presentation of peptide or polysaccharide antigen via MHC class II*, *phagocytosis* and marker of the *hematopoietic cell lineage*. Additionally, we observed a raised expression of the monocytic maturation marker *CD14*, *CEBPB*, *EGR2*, *FOS*, *MAFB*, *MNDA* and *MHC class II* as well as a reduced expression of markers of immature cells of the monocytic lineage (*ELANE* and *CTSG*) [24-27].

A high number of transcriptional regulators (38 genes) were affected by MLL-AF9 depletion. Several (i.e. *CEBPB*, *FOS* and *FOSB*) were also described to be deregulated in the transition of CML blastic phase to an acute leukemia [23].

Calcium associated genes as well as genes involved in *response to estrogen stimulus* were strongly enriched after MLL-AF9 depletion, implying an influence of MLL-AF9 on calcium ion homeostasis and estrogen response effects. The enriched annotation *chaperone binding* contains proteins which interact selectively and non-covalently with a chaperone protein and encompassed four down-regulated genes in our data set. The enrichment of genes within the annotation *regulation of cell size* is consistent with our phenotypic findings and encompassed the genes *AR*, *ATP2B2*, *EMP1*, *LAMB2*, *NOTCH2*, *PLXNA3*, *SGMS1*, *TGFBR1* and *WFDC1*.

The higher-order term *early development* suggests downstream effects of MLL-AF9 analogous to MLL and AF9 wildtype proteins. This concerns effects on pattern specification processes, mainly via homeobox genes. Other enriched annotation terms suggested an influence of MLL-AF9 on genes involved in *aging* and on genes encoding proteins of the *actin cytoskeleton* and the *extracellular matrix*.

GSEA analysis for our entire gene expression profile (which does not employ a significance cutoff value) additionally suggested effects on DNA repair, MAP kinase activity, RNA polymerase activity, splicing, ribosomal constituents and translation (Additional file 2).

We further asked which of the functional processes influenced by MLL-AF9 are due to direct effects of MLL-AF9. For this purpose, we performed a gene ontology enrichment analysis (DAVID) for previously published direct targets of MLL-AF9 derived from a mouse model [13] (Additional file 4) and compared the results to our gene ontology data. The overlap between the data sets suggests that transcriptional regulators such as those involved in early development (homeodomain genes) are mainly direct MLL-AF9 targets. On the other hand, the majority of processes we identified as affected by MLL-AF9 depletion (like apoptosis, calcium and estrogen related genes and monocyte/macrophage differentiation) are likely governed by indirect effects of MLL-AF9.

### *MLL-AF9* knockdown is associated with reduced cell size

**Figure 4:**
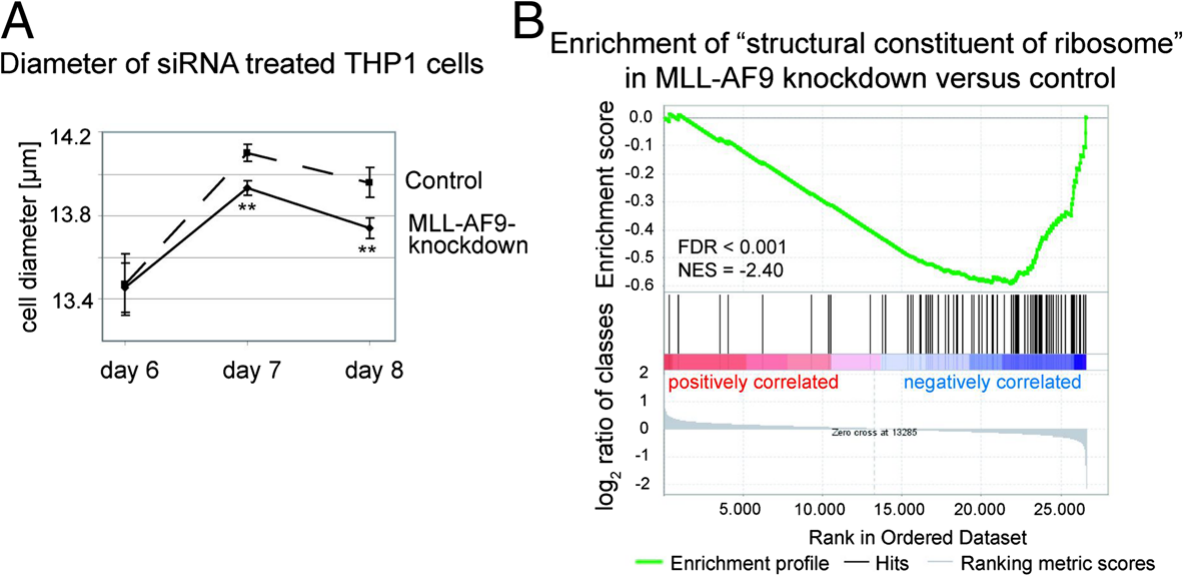
**Effects of *MLL-AF9 *knockdown in THP1 cells related to cell size. (A)** Mean cell diameter between *MLL-AF9* knockdown and non-targeting siRNA control cells was significantly altered on day 7 and 8 of experiments. Graph represents data from five independent experiments. Bars indicate standard deviation. **p < 0.005. **(B)** Gene set enrichment plot of genes from the gene ontology term “structural constituent of ribosome” (GO:0003735) for our gene expression profile after *MLL-AF9* knockdown in THP1 cells. FDR, false discovery rate; NES, normalized enrichment score.

We did not observe effects of MLL-AF9 depletion on proliferation, cell cycle distribution and apoptosis rate of THP1 cells (Additional file 3: Figure S2). However, we consistently detected a significant reduction of 0.2 μm in mean cell diameter between *MLL-AF9* knockdown and control treatments (Figure 4A). MLL-AF9 specific reduction in cell size was evident under serum reduced conditions and prolonged *MLL-AF9* knockdown but not in the presence of 10% FCS (up to day 8) or upon a shorter time frame (up to day 3).

Cell size has previously been described to be closely linked to ribosomal biogenesis [28]. GSEA indeed revealed genes encoding structural constituents of ribosomes to be significantly enriched in down-regulated genes after *MLL-AF9* knockdown in THP1 cells (Figure 4B). The core enrichment gene set was represented by 42 ribosomal proteins of small and large subunits of both, cytoplasmic and mitochondrial ribosomes.

### Target genes likely mediating MLL-AF9 leukemogenic effects

**Figure 5:**
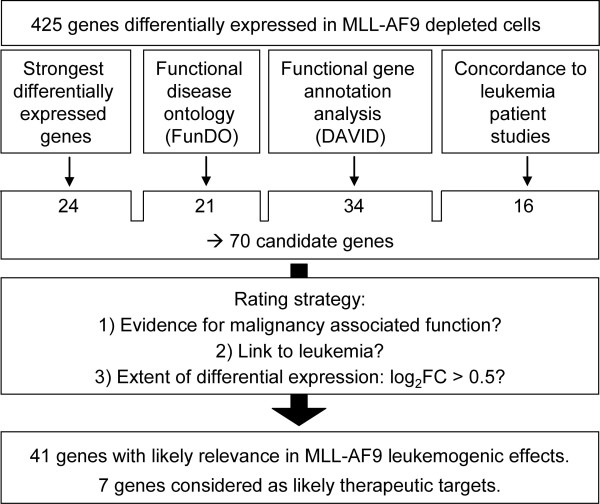
**Strategy to prioritize candidate genes concerning mediation of MLL-AF9 leukemogenic effects.** Four approaches (second line) were used to select 70 genes from the set of 425 differentially expressed genes after *MLL-AF9* knockdown in THP1 cells. Each gene’s likelihood to play a role in leukemogenesis was rated via a structured rating strategy based on data obtained through a literature search.

**Figure 6:**
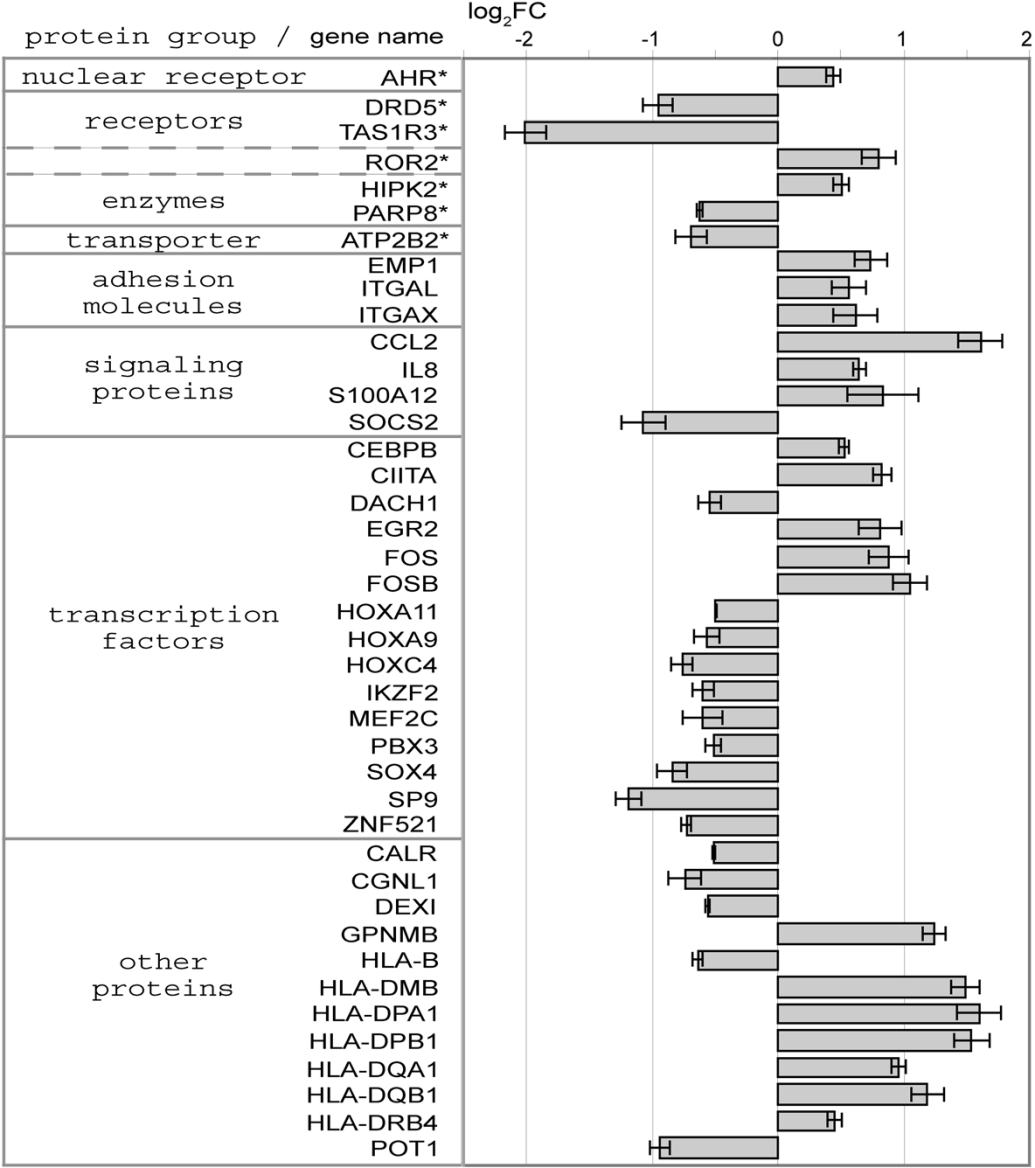
**Differential expression after *MLL-AF9 *knockdown of genes considered as likely mediators of MLL-AF9 leukemogenic effects.** Columns show log_2_FC from microarray data of *MLL-AF9* knockdown versus control treatments on day 8. Standard error of the mean is indicated. Transcripts are sorted according to categories of encoded proteins and alphabetically. Dotted division lines indicate proteins assorted to two categories. *Genes coding for proteins which belong to conventionally druggable protein classes as described by Rask-Andersen et al. [11].

Among the 425 differentially expressed genes, 41 candidates for mediation of MLL-AF9 leukemogenic effects were prioritized by a stepwise approach (Figure 5). Selected were (1) genes with strongest differential expression (≥ ± 1.0 log_2_ fold change (log_2_FC)), (2) leukemia associated genes defined by functional disease ontology (FunDO), (3) the strongest regulated (≥ 1.0 log_2_FC and further top 5) genes of each higher-order term from functional gene annotation results and (4) genes that showed concordant differential expression between our *in vitro* data and published leukemia patient studies. Due to overlapping hits, this added up to 70 genes which were subsequently subjected to literature research and rated via a structured strategy (Additional file 3: Table S2). This approach resulted in 41 genes being rated as likely mediators of leukemogenic effects of MLL-AF9 (Figure 6).

Receptors, including nuclear receptors (i.e. ligand-activated transcription factors), enzymes and transporter have yielded especially successful therapeutic targets for clinical intervention [11]. Thus, we judged those among our 41 top rated genes which encode receptors, enzymes or transporter as potential therapeutic targets downstream of MLL-AF9. An overview of the relevant biological roles of these seven potential therapeutic targets, including the dopamine receptor DRD5, is presented in Table 2.

**Table 2:** Genes deregulated by MLL-AF9 and prioritized as potentially druggable targets in MLL-AF9 leukemia

**Gene**	**log**_**2 **_**FC**	**Protein class**	**Official full name**
			**Biological role**
AHR	0.44	Nuclear receptor	Aryl hydrocarbon receptor
			Upregulated by AML associated fusion gene AML1-ETO. Differentiation of myeloblastic leukemia cells. Estrogen receptor degradation. AHR knockout mice display CML.
ATP2B2	−0.69	Transporter	ATPase, Ca++ transporting, plasma membrane 2
			Lowers intracellular calcium; protects from apoptosis.
DRD5	−0.96	Receptor	Dopamine receptor D5
			Raised after G-CSF treatment; dopamine receptor agonists activate Wnt signaling, induce migration and increase clonogenic capacity and repopulation of CD34+ cells.
HIPK2	0.5	Enzyme	Homeodomain interacting protein kinase 2
			Phosphorylates transcription (co-) factors (e.g. c-Myb); may trigger (myeloid) differentiation and apoptosis. Mutations found in AML cases.
PARP8	−0.63	Enzyme	Poly (ADP-ribose) polymerase family, member 8
			Phosphorylated upon DNA damage. Upregulated in *MLL* rearranged AML patients.
ROR2	0.81	Receptor/enzyme	Receptor tyrosine kinase-like orphan receptor 2
			Mediates noncanonical Wnt signaling. Putative tumor suppressor in leukemia, presumably via inhibition of Wnt canonical signaling.
TAS1R3	−2.01	Receptor	Taste receptor, type 1, member 3
			Glucose absorption/energy supply. Heterodimers sense extracellular amino acids, activate MTORC1 and inhibit autophagy.

Details to biological roles and references are given in Additional file 3: Table S2. Log_2_FC: log_2_ fold change in MLL-AF9 knockdown relative to control.

### The dopamine receptor antagonist SCH39166 exerts effects on malignant cell characteristics of THP1 cells

As a proof of principle for the functional relevance of the suggested therapeutic targets, we examined the effects of the DRD1- and DRD5-specific dopamine receptor antagonist SCH39166 (ecopipam) on THP1 cells.

**Figure 7:**
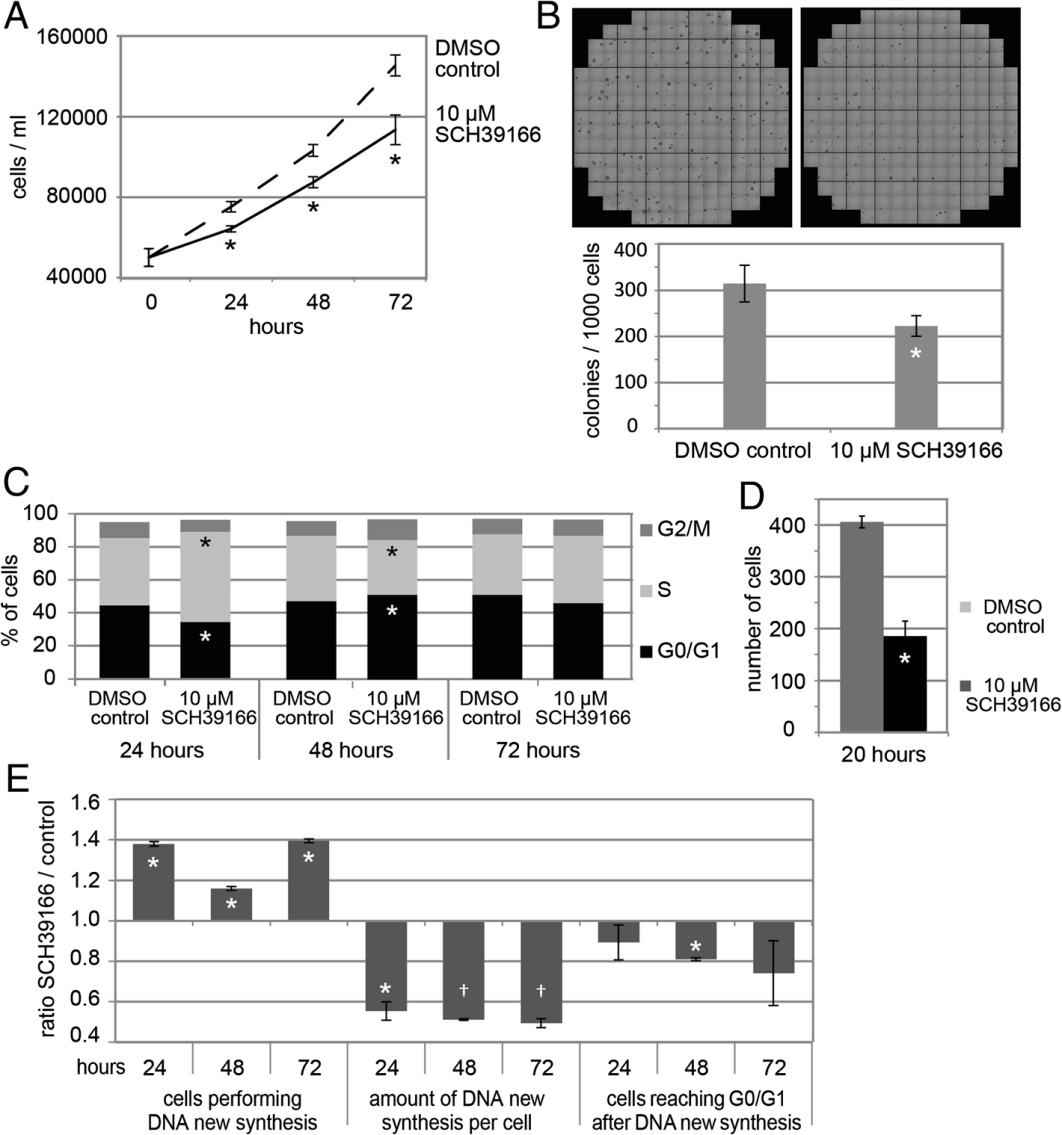
**THP1 cell characteristics influenced by 10 μM of the dopamine receptor antagonist SCH39166 compared to DMSO control. (A)** Proliferation in serum reduced conditions. **(B)** Colony formation in methyl cellulose. Images show representative wells of DMSO control (left) and 10 μM SCH39166 treatment (right). **(C)** Cell cycle distribution in serum reduced conditions. **(D)** Cell migration in 5 μm transwells. **(E)** DNA new synthesis rate measured via EdU incorporation and SYTOX AADvanced nuclear stain. Ratio of SCH39166 compared to DMSO control treatment is shown. Mean over three **(A-C)** or two **(D-E)** independent experiments are shown. Bars indicate standard error of the mean; *p < 0.05. † Non significant differences because of interexperimental fluorescence intensity standard deviation, although the ratio between the two treatments remains similar.

Treatment of THP1 cells with 10 μM SCH39166 led to a number of significant changes in cell characteristics: (1) proliferation and (2) colony forming capacity were reduced, (3) cell cycle analysis revealed alternating changes in G1 and S phase distributions which may suggest a slowdown of cell cycle progression, (4) cells were slower to reach G0/G1 phase after DNA new synthesis which was reduced and (5) the cell migration rate was decreased (Figure 7). No increase in the rate of apoptotic THP1 cells was observed (Additional file 3: Figure S3).

## Discussion

The experimental setup of this RNA interference study was optimized to exclude off-target effects and to ensure a specific reduction of *MLL-AF9* protein on functional level. Among seven human cell lines carrying the MLL-AF9 translocation, the cell line THP1 was chosen for this study because it is the only one established from a characteristic infant AML M5 leukemia patient [29]. According to genomic DNA gains and losses as well as gene expression, THP1 – like other leukemia cell lines – has been described to be a faithful model system for leukemia with genomic aberrations [30]. As compared to overexpression studies, RNAi enabled us to modify MLL-AF9 at physiological range and to target all putative transcript variants of MLL-AF9 which retain the breakpoint exons.

The validity of the generated differential gene expression profile as well as the suitability of our experimental approach was supported by the significant enrichment of several gene sets generated by previous studies: *Leukemia* was the top ranked associated disease term, and direct MLL-AF9 murine targets [13] as well as MLL-AF9 downstream genes detected in transduced neonatal and myeloid as well as lymphoid human primary cells [14] were significantly enriched. These findings validate our experimental approach in that expected information content was indeed disclosed in our data. It also indicates that our *MLL-AF9* knockdown model represents neonatal and mixed lineage features as expected for *MLL-AF9* positive leukemia.

Additionally, concordance of our *in vitro* model system to the *in vivo* situation was suggested by enrichment of *MLL* associated gene signatures from various myeloid leukemia patient studies. Among the enriched signatures were those of four AML patient studies but also one data set characterizing CML leukemia blastic phase as compared to chronic phase [23]. As this step marks the transition to an acute leukemia [23], the overlapping genes (*CEBPB*, *FOS*, *FOSB*, *IL8*, *S100A12*, *SOCS2*) might be involved in the aggressiveness of *MLL-AF9* myeloid leukemia blasts.

To characterize biological processes influenced by MLL-AF9, we performed functional studies in our experimental system and employed functional gene ontology analyses on our MLL-AF9 modulated gene expression profile which encompassed 425 differentially expressed genes.

Most remarkable in our functional results was a significant reduction of cell size in MLL-AF9 depleted THP1 cells. Enriched differential expression of genes within the annotation *regulation of cell size* (e.g. *ATP2B2* and *EMP1*) was consistent with this observation. The annotation terms *structural constituents of ribosomes* and *cellular calcium ion homeostasis* were also linked to our data set and could contribute to the observed cell size reduction. Ribosomal biogenesis is a known important determinant of cell size [28] and has been linked to tumorigenesis and malignancies [31], while intracellular Ca^2+^ levels can regulate reactive volume changes of cells via activation of K^+^ and Cl^-^ channels [32].

Enrichment of annotation terms related to proliferation and apoptosis implied that MLL-AF9 is involved in replication and cell death via downstream transcripts. Previous studies have detected effects of MLL-AF9 on these cellular characteristics after treatment with antisense phosphorothioate-oligodeoxyribonucleotides [33,34]. In our hands, however, neither the proliferation rate nor the proportion of apoptotic THP1 cells was detectably altered after *MLL-AF9* knockdown. This might be due to favorable *in vitro* growth conditions and the absence of sufficient apoptosis stimuli in our cell culture situation. Nevertheless, the differential gene expression signature generated in this study yielded genes which can influence apoptosis and might contribute to the mitogenic phenotype of *MLL-AF9* positive monoblasts in a less ideal environment possibly present in the *in vivo* disease state. These genes encode for example POT1 which promotes telomere elongation [35], CALR which promotes DNA synthesis and inhibits growth arrest and senescence (via p21) [36] and HIPK2 which is involved in induction of cell death and differentiation [37].

*MLL-AF9* is regarded as a class II mutation, primarily interfering with cellular differentiation [38]. In line with this, a major effect of MLL-AF9 on monocytic differentiation was also revealed by our data: Numerous significantly enriched, functional annotation terms concerned fundamental functions of monocytes and macrophages. Moreover, the differential expression of nine marker genes clearly suggested maturation of MLL-AF9 depleted THP1 monoblasts. This implies that the MLL-AF9 induced differentiation stop is, at least in part, reversible. It is thus tempting to speculate that therapeutic differentiation strategies as successfully employed for acute promyelocytic leukemia [39] might also be feasible for *MLL* aberrant AML.

The enriched annotations r*esponse to estrogen stimulus* and *chaperone binding* might also be related to leukemogenic mechanisms. MLL-AF9 might inhibit estrogen receptor degradation and retinoic acid signaling pathways leading to impaired cellular differentiation: AHR and MAPK15 which are reported to target estrogen receptor alpha to degradation [40,41] were reduced in their expression by MLL-AF9 in our study. Estrogen and retinoic acid signaling are known to regulate gene expression in opposing directions [42]. Furthermore, promoter methylation (indicating transcriptional repression) of estrogen receptor is associated with increased survival of AML patients and reduced in AML M5 [43]. Chaperones have been linked to deregulated cell growth as forced overexpression of chaperones was shown to result in cellular transformation and tumor formation [44]. Within our data set, all four *chaperone binding* genes were down-regulated. One of these, calreticulin, encodes a multifunctional protein which acts also as chaperone and is known to inhibit the translation of *CEBPA*, a key myeloid transcription factor frequently disrupted in AML [45].

A profound effect of MLL-AF9 on downstream gene expression was suggested by significantly enriched annotations encompassing 38 differentially expressed transcription factors. One example is the up-regulation of *CIITA*, the master coactivator of MHC class II [46]. Accordingly, in MLL-AF9 depleted THP1 cells, we observed an up-regulation of all MHC class II genes (HLA-DP, -DQ, -DR, -DM) with exception of the non-classical HLA-DO which seems less dependent on the CIITA transcription factor [46]. Up-regulation of HLA class II gene transcripts has previously been associated with differentiation of monoblasts, while THP1 cells were observed to have reduced HLA class II expression levels compared to mature monocytes [24].

Results from functional annotation analysis indicated that MLL-AF9’s direct targets are predominantly transcriptional regulators while other processes we identified, like monocyte/macrophage differentiation or estrogen response are likely related to indirect effects of MLL-AF9. Although considerable progress has been made, transcription factors have been found difficult to target therapeutically [47]. This emphasizes the importance of determining indirect targets downstream of MLL-AF9 to identify MLL-AF9 leukemogenic effectors amenable to be addressed by small molecule drugs.

We are aware that our prioritization strategy to select candidate targets is not all-inclusive. Some candidates may be lost during the primary subset selection or by our rating procedure due to a lack of specific prior knowledge on these gene products. Our comprehensive gene expression data set (GEO: GSE36592) may thus hold a yet vaster source of informative content. Nevertheless, we prioritized 41 candidates potentially involved in mediation of MLL-AF9 leukemogenesis. Among these are many novel as only 26 were identified in previous *MLL-AF9* studies [3,13,14,48] and only one, HOXA9, is a known direct target of MLL-AF9. Some of our candidates are potential therapeutic targets, primarily those encoding receptors, enzymes, and transporter: These classes represent almost 90% of all therapeutic effect-mediating human protein targets of drugs listed by the US Food and Drug Administration (FDA) [11]. Seven of our top-41 candidates encode proteins of these classes: AHR, ATP2B2, DRD5, HIPK2, PARP8, ROR2 and TAS1R3.

As a proof of principle, we selected one of these suggested targets, the dopamine receptor DRD5, for further investigation. Expression of DRD5 is raised in CD34+ hematopoietic progenitor cells after G-CSF treatment and dopamine receptor agonists activate Wnt signaling, induce migration and increase clonogenic capacity and repopulation in this cell type [49].

We employed the drug SCH39166 (ecopipam) which is currently one of the most specific antagonists for the dopamine receptor DRD5 and antagonizes DRD1 and DRD5 receptors but not receptors of the DRD2-class. Treatment of THP1 cells with SCH39166 exerted a number of significant anti-malignant effects, i.e. affecting proliferation, clonogenic capacity, cell cycle distribution, DNA new synthesis and cell migration.

The safety profile of SCH39166 has been studied in over 1000 people and SCH39166’s ability to ameliorate multiple central nervous system diseases is under investigation in clinical phase 1–3 trials (Psyadon Pharmaceuticals, Inc.). In a large compound screening study, Sachlos et al. recently discovered that thioridazine - which antagonizes the dopamine receptors 1 to 5 – selectively targets neoplastic cells and human leukemic stem cells. In contrast, no effects of thioridazine were reported on normal blood stem cells which do not express dopamine receptors [50].

Previous data also indicate an influence of some of our suggested therapeutic targets e.g. on cellular differentiation, Wnt signaling and apoptosis (Table 2). The nuclear receptor AHR e.g. has been shown to promote retinoic acid–induced differentiation of HL-60 myeloblastic leukemia cells, if overexpressed [40] while AHR knockout mice are diagnosed with CML [51]. DrugBank search results [52] revealed that AHR and DRD5 interact with 6 and 18 approved drugs respectively, although developed for other medical indications. The candidates ATP2B2, PARP8 and TAS1R3 have previously not been functionally linked to leukemia.

The present study provides a comprehensive gene expression profile after *MLL-AF9* knockdown in THP1 cells that broadens our insights into the molecular mechanisms and druggability of *MLL* aberrant leukemia. The finding of a more differentiated state of THP1 cells after MLL-AF9 depletion is consistent with the hypothesis that the MLL-AF9 induced differentiation stop is reversible. We describe here more than 40 gene products and several cellular processes likely involved in mediation of leukemogenic effects of MLL-AF9. Among these, seven targets were classified as potentially druggable and targeting one of these, DRD5, shows anti-leukemic effects in THP1 cells. Altogether, our results might support the search for new targeted therapies for *MLL-AF9* positive pediatric AML.

## Methods

### Cell cultivation

THP1 cells (DSMZ GmbH, Braunschweig, Germany) were maintained in RPMI1640, 10% heat-inactivated fetal calf serum (FCS), 100 U/ml Penicillin and 0.1 mg/ml Streptomycin (PAA Laboratories, Pasching, Austria) at a density of 0.05-0.5 × 10^6^ cells/ml. To minimize the effect of FCS on growth related and mitogenic signaling pathways, serum levels were reduced from experimental day 6 onwards. Serum reduced conditions were achieved by washing cells in PBS and resuspending them in DMEM/Ham’s-F12 medium containing 1 g/L BSA and 0.5% Fetal Bovine Serum (Gibco, Life Technologies, Carlsbad, CA, USA). For functional studies of DRD5, THP1 cells were treated with 10 μM SCH39166/ecopipam (Tocris Bioscience, Bristol, UK) or DMSO as control.

### Small interfering RNA and transfection

Silencer Select siRNAs (siRNA-A, siRNA-B, negative control siRNA-1 and −2, Ambion, Life Technologies) were dissolved in 1 x siRNA buffer (Dharmacon, Lafayette, CO, USA). SiRNA-A and -B sequences are shown in Figure 1A. Transfections were performed according to the supplier’s protocol with 9 μl Dreamfect (OZ Biosciences, Marseille, France) at a final concentration of 50 nM siRNA within 1 ml culture medium that contained 5 × 10^4^ cells in a 12-well format (Greiner, Kremsmuenster, Germany). Transfection efficiency was measured by flow cytometry with fluorescently labeled control siRNA.

Experimental incubations lasted eight days with repeated transfections on day 0, 3, and 6. Prior to each transfection event, cell densities were determined by Cellscreen System (Innovatis, Bielefeld, Germany) on an Olympus IX 50 microscope (Olympus, Tokyo, Japan) and cells were subsequently reseeded at 5 × 10^4^ cells per ml.

### RNA isolation and reverse transcription quantitative PCR

Total RNA was extracted with miRNeasy Mini Kit (Qiagen, Hilden, Germany) according to the supplier’s protocol. Reverse transcription quantitative PCR (RT-qPCR) was carried out in triplicates with 600 ng total RNA, QuantiTect Reverse Transcription Kit (Qiagen) and iQ™-SYBR® Green Supermix (Biorad, Hercules, CA, USA) on a StepOnePlus instrument (Applied Biosystems, Life Technologies) according to the supplier’s protocol. *RPL13A* and *UBC*, showing stable expression in bone marrow [53], were used as reference genes. Data were analyzed with StepOne software v 2.1 (Applied Biosystems) and the ∆∆C_q_ method. Primer sequences are provided in Additional file 3: Table S3.

### Immunoblotting

Total cellular protein was recovered from Qiazol organic phase according to TRI reagent protein isolation protocol (Ambion), resuspended in 9.5 M urea, 4% [w/v] CHAPS, mixed 1:1 with Laemmli sample buffer (2×, Sigma-Aldrich, St. Louis, MO, USA) and incubated 5 minutes at 95°C. Proteins were separated by SDS-PAGE and transferred onto PVDF membrane in transfer buffer (10 mM CAPS, pH 11, 10% methanol, 0.01% SDS). Membranes were probed with anti-MLL1, anti-AF9 (A300-086A, A300-597A, Bethyl Laboratories, Montgomery, TX, USA), anti-Lamin A/C and secondary antibodies (sc-7292, sc-2004, sc-2005, Santa Cruz Biotechnology, Santa Cruz, CA, USA) followed by detection using ECL-substrate. Band intensities were analyzed with ImageJ version 1.46r (National Institutes of Health, Bethesda, MD, USA).

### Gene expression profiling analysis

Human Whole Genome Microarrays 4x44K v2 (Agilent Technologies, Santa Clara, CA, USA) were commissioned to and performed at IMGM Laboratories (Martinsried, Germany). Two knockdown (siRNA-A, siRNA-B) and two negative control siRNA samples, each containing 100 ng pooled RNA of five independent experiments were utilized. RNA concentration and purity (abs 260/280 nm) were analyzed using a Nanodrop ND-1000 Spectrophotometer (Nanodrop Technologies, Wilmington, DE, USA). RNA integrity was determined with an RNA 6000 Nano LabChip Kit on a 2100 Bioanalyzer (Agilent Technologies). A260/A280 was above 2.0 and RNA integrity number was above 9.3.

Hierarchical clustering of arrays and principal component analyses were performed with the statistical language R version 2.11.1 [54] using packages from the Bioconductor framework [55]. Analysis of differential expression was carried out in R with the limma package [56,57]. Adjustment for multiple testing was done using the method by Benjamini and Hochberg [58]. For differential expression analysis, the unnormalized data were favored over the normalized as they showed an almost perfect and superior logarithmic intensity distribution. Normalized values showed no improved principal component analysis and hierarchical clustering concerning similarity of biologically related arrays. Unnormalized identical replicate probes yielded more consistent intensity values compared to the normalized values which further supported this approach. Probes were considered as differentially expressed at a *p*-value of the moderated *t* test below 0.005 and significant alteration over all identical replicate probes (*t* test, p < 0.05).

To test reliability of microarray data, RT-qPCR was performed for 14 arbitrarily selected transcripts which showed differential expression in microarray results (ARHGAP26, CALR, CEBPB, CIITA, FOS, FUCA1, MAFB, NOTCH2, SOCS2, SULF2, TSPAN14, VASH1, VDR and ZNF521). Spearman’s rank correlation coefficients including *p* values were calculated with R. Spearman’s *Rho* was 0.91 (*p* 5.15 × 10^-6^) for technical replicates (identical RNA samples in RT-qPCRs and microarrays) and 0.72 (*p* 0.004) for biological replicates (RT-qPCRs performed on RNA sample pools of 2 independent experiments).

### Functional bioinformatics

Functional disease ontology (FunDO) [59] and functional annotation analysis (Database for Annotation, Visualization and Integrated Discovery version 6.7, DAVID) [19] was performed with input of all 425 differentially expressed genes. Enrichment was regarded as significant if fold enrichment was ≥ 1.5 and *p*-value < 0.1. Functional annotation analysis of direct targets of MLL-AF9 was performed with a previously published gene list [13] transformed to the official gene symbols. Gene set enrichment analysis was executed with the computational method GSEA version 2.0 [60,61]. Gene sets associated to leukemia were extracted from FunDO and from leukemia patient studies [1,20-23] and transformed to the official gene symbols. Parameter settings were default except for: dataset was not collapsed to gene symbols, instead the strongest regulated probe per gene was selected; *gene set* was selected as permutation type; metric for ranking genes was *log2 ratio of classes*; gene list sorting mode was either *real* for gene sets including only up- or down-regulated genes or *abs* for gene sets including up- and down-regulated genes. A rating strategy for prioritization of genes likely mediating MLL-AF9 leukemogenic effects was devised as outlined in Additional file 3: Table S4.

### Biological assays

Proliferation and cell diameter were measured via Cellscreen System (Innovatis) on an Olympus IX50 microscope. This microscopic monitoring system provides fully automated non-invasive cell count in cell culture well plates by generating microscopic images at defined regions of interest (ROIs). By digital image recognition, cells are automatically detected, counted and their geometry (diameter and eccentricity) is analyzed [62]. Measurements were performed in 12-well plates (Greiner) with 62 ROIs per well equivalent to ~ 5 000 - 10 000 counted cells. Cells were allowed to settle for 20 minutes prior to measurement. A Welch’s *t* test was performed over replicate experiments after confirmation of normal distribution via Shapiro-Wilk normality test.

Cell cycle analyses were performed via flow cytometry. Cells were fixed in 70% ethanol at −20 °C, subsequently stained for 30 min with propidium-iodide (0.1% Triton X-100, 0.2 mg/ml RNase A, 20 μg/ml propidium-iodide in PBS) and analyzed on a BD FACSCanto™ (BD Biosciences, Franklin Lakes, NJ, USA). Cell doublets and aggregates were removed by gating and the proportion of cells in G0/G1, S and G2 phase were quantified with Watson Pragmatic model in FlowJo 9.7.2. DNA new synthesis rate was measured by flow cytometry after pulse labeling THP1 cells with 25 μM EdU (5-ethynyl-2’-deoxyuridine) for 2 hours and staining with AF488-azide and SYTOX AADvanced nuclear stain (Invitrogen, Life Technologies) according to supplier’s protocol. Statistics were calculated with Shapiro-Wilk normality and Welch’s *t* test in R.

Apoptotic rate was analyzed via intracellular staining of cleaved PARP1 [63]. Cells were fixed and permeabilized with Foxp3 Staining Buffer Set (eBioScience, San Diego, CA, USA) and stained with anti-PARP antibody (44–699, Invitrogen) after blocking with 5 μg human IgG (Sigma).

Colony forming capacity was analyzed by plating 1000 THP1 cells in 1 ml 0.5% methyl cellulose (64630, Sigma)/RPMI1640 (10% FCS) in 6-well plates. Colonies were counted in microscopic images taken after 10 days by Cellscreen system.

Migration was analyzed in a transwell assay (5 μm pore size, #3421, Corning, NY, USA) with 0.1 × 10^6^ THP1 cells in RPMI1640 / 10% FCS in upper chamber and RPMI1640 with 10% FCS and 0.1 mM ascorbic acid in lower chamber. Cells in lower chamber were counted in microscopic images taken after 20 hours by Cellscreen system.

For analyses of SCH39166 effects, cells were pre-treated with drug or DMSO for 3 hours prior to performing assays.

## Competing interests

The authors declare no conflict of interest.

## Authors’ contributions

KKF designed the project, conceived and performed the experiments, analyzed and interpreted the data and wrote the manuscript. PP provided analytical tools, analyzed the data and critically revised the manuscript. IS interpreted the data, provided critical discussion and critically revised the manuscript. AAR designed the project, conceived experiments, wrote the manuscript and supervised the project. All authors read and approved the final manuscript. There are no potentially redundant publications.

## Supplementary Material

Additional file 1 **Differentially expressed probes in MLL-AF9 knockdown.** Listed are all differentially expressed probes (p < 0.005) from gene expression arrays of MLL-AF9 depleted versus control THP1 cells on day 8 of experiments. Click here for file

Additional file 2 **Gene set enrichment analysis (GSEA) results.** (Sheet 1) GSEA of gene sets identified by Horton et al. [14] employing *MLL-AF9* transduced primary human cells for the *MLL-AF9* knockdown gene expression profile of this study. “Up” or “down” indicates gene sets comprising either up- or down-regulated genes after MLL-AF9 transfection of cord blood (neonatal) or bone marrow (adult) CD34+ cells by Horton et al. [14]. (Sheet 2) GSEA of gene ontology (GO) terms for the gene expression profile after *MLL-AF9* knockdown in this study (false discovery rate < 0.05). Click here for file

Additional file 3: Figures S1-S3 and Tables S1-S4 Figure S1. GSEA enrichment plots of published leukemia patient studies for our *MLL-AF9* knockdown gene expression profile. Table S1: Assignment of enriched functional annotation terms to higher-order terms. Figure S2: Cell characteristics of MLL-AF9 depleted and control THP1 cells. (A) Proliferation. (B) Cell cycle distribution. (C) Apoptosis rate. Figure S3: Apoptosis rate in THP1 cells treated with 10 μM SCH39166 compared to DMSO control. Table S2: Biological roles and rating of 70 selected genes differentially expressed after MLL-AF9 knockdown. Table S3: Primer used for reverse transcription quantitative PCR. Table S4: Rating strategy to prioritize candidate targets for mediation of MLL-AF9 leukemogenic effects. A total of 70 differentially expressed genes after MLL-AF9 knockdown was rated. Click here for file

Additional file 4 **Results of gene ontology analyses performed via DAVID software.** Terms were regarded as significantly enriched if fold enrichment was ≥ 1.5 and p value ≤ 0.1. (Sheet 1) Significantly enriched annotation terms which were regarded as potentially relevant in this biological setting. Terms were identified via DAVID analysis of our gene expression profile after MLL-AF9 knockdown. Table includes the differentially expressed genes of each term. (Sheet 2) All significantly enriched functional gene ontology terms identified for our gene expression profile after MLL-AF9 knockdown. (Sheet 3) Significantly enriched functional gene ontology terms for direct MLL-AF9 targets identified in a mouse model by Bernt et al. [13]. Click here for file
