# Supplementary material for: RNAi-mediated silencing of MLL-AF9 reveals leukemia-associated downstream targets and processes
Source: Mol Cancer. 2014 Feb 11;13:27. doi: 10.1186/1476-4598-13-27 (PMC3924703; doi:10.1186/1476-4598-13-27)
Supplement: Additional file 3: Figures S1-S3 and Tables S1-S4 — Figure S1. GSEA enrichment plots of published leukemia patient studies for our MLL-AF9 knockdown gene expression profile. Table S1: Assignment of enriched functional annotation terms to higher-order terms. Figure S2: Cell characteristics of MLL-AF9 depleted and control THP1 cells. (A) Proliferation. (B) Cell cycle distribution. (C) Apoptosis rate. Figure S3: Apoptosis rate in THP1 cells treated with 10 μM SCH39166 compared to DMSO control. Table S2: Biological roles and rating of 70 selected genes differentially expressed after MLL-AF9 knockdown. Table S3: Primer used for reverse transcription quantitative PCR. Table S4: Rating strategy to prioritize candidate targets for mediation of MLL-AF9 leukemogenic effects. A total of 70 differentially expressed genes after MLL-AF9 knockdown was rated. [file 1476-4598-13-27-S3.pdf]

Supplementary information to "Fleischmann KK, Pagel P, Schmid I, Roscher AA. RNAi-mediated silencing of MLL-AF9 reveals leukemia-associated downstream targets and processes".

Figure S1: GSEA enrichment plots of published leukemia patient studies for our *MLL-AF9* knockdown gene expression profile. Enrichment analyses of down-regulated gene sets (d, g) were analyzed for our profile divided in up- and down-regulated genes, while genes sets including up- and down-regulated genes (a-c, e-f) were analyzed for our profile of deregulated genes in general (absolute log_2FC values). CML-BP, chronic myeloid leukemia blastic phase; CML-CP, chronic myeloid leukemia chronic phase; FDR, false discovery rate q-value; NES, normalized enrichment score.

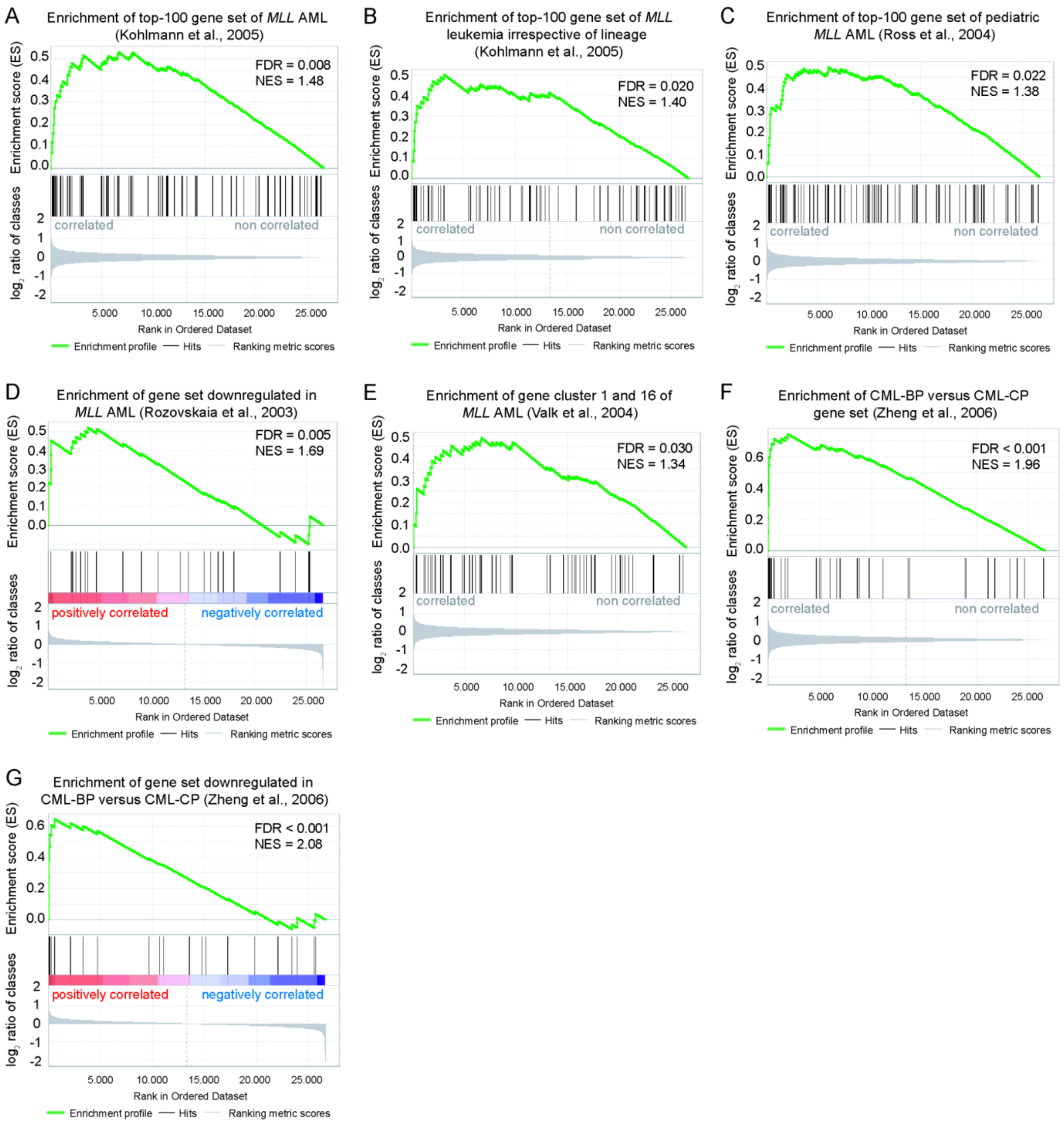

Table S1: Assignment of enriched functional annotation terms to higher-order terms. Terms were identified via DAVID analysis of our gene expression profile after MLL-AF9 knockdown in THP1.

**Higher-order term**	**Functional annotation term (DAVID)**	**Rationale**
proliferation and apoptosis	regulation of DNA replication	DNA replication is a required step in proliferation.
proliferation and apoptosis / monocyte/macrophage differentiation	protein kinase C activity	Protein kinases C (PKC) function either as activators or inhibitors of apoptosis, depending on the particular cell type and the specific apoptotic stimulus [1]. Increased PKC activity in monocytes enhances their initial adhesion and their differentiation into macrophages [2].
proliferation and apoptosis / monocyte/macrophage differentiation	response to wounding	This functional process is associated with cell proliferation involved in wound healing, as well as with inflammatory responses. Monocytes are known to migrate into wound regions where they are involved in removing tissue debris and secreting factors that promote growth and biosynthetic activity of fibroblasts [3].
proliferation and apoptosis / monocyte/macrophage differentiation	JAK-STAT cascade	JAK activation stimulates cell proliferation, differentiation, migration and apoptosis [4].
proliferation and apoptosis / monocyte/macrophage differentiation	cytokine binding	Term defined as: „Interacting selectively and non-covalently with a cytokine, any of a group of proteins that function to control the survival, growth and differentiation of tissues and cells, and which have autocrine and paracrine activity.“
monocyte/macrophage differentiation	antigen processing and presentation	Antigen processing and presentation via MHC class II is a fundamental function of professional antigen presenting cells like monocytes and macrophages.
monocyte/macrophage differentiation	antigen processing and presentation of peptide or polysaccharide antigen via MHC class II	
monocyte/macrophage differentiation	positive regulation of immune system process	Monocytes are cells of the innate immune response.
monocyte/macrophage differentiation	defense response	
monocyte/macrophage differentiation	response to bacterium	

Higher-order term	Functional annotation term (DAVID)	Rationale
monocyte/macrophage differentiation	leukocyte chemotaxis	Monocytes and macrophages produce chemokines (e.g. IL8) that attract other leukocytes to a site of infection.
monocyte/macrophage differentiation	phagocytosis	Monocyte / macrophages are involved in the scavenging of dying cells, pathogens, and molecules through phagocytosis and endocytosis [5].
monocyte/macrophage differentiation	endocytosis	
monocyte/macrophage differentiation	phorbol ester binding	Phorbol esters (such as PMA) are inducers of monocyte / macrophage differentiation in many myeloid cell lines [6].
monocyte/macrophage differentiation	lysosome	Monocytes are responsible for phagocytic uptake and digestion of microbes and particles (via lysosomes).
monocyte/macrophage differentiation	Hematopoietic cell lineage	This annotation term refers to stage- and lineage-specific surface markers of hematopoiesis.
monocyte/macrophage differentiation / early development	Homeobox, conserved site	Homeobox domain-containing proteins (encoded e.g. by Hox genes) are transcriptional regulators which are essential for correct positioning of segmented structures in early embryogenesis, while they also play a role in normal proliferation and differentiation of hematopoietic stem and progenitor cells [7].
early development	pattern specification process	This term is defined as "any developmental process that results in the creation of defined areas or spaces within an organism to which cells respond and eventually are instructed to differentiate".

Six functional annotation terms were directly related to the higher-order term and are not listed (positive / negative regulation of apoptosis, transcription factor activity, regulation of transcription from RNA polymerase II promoter, cellular calcium ion homeostasis, calcium).

References to Table S1:

1. Voss OH, Kim S, Wewers MD, Doseff AI: **Regulation of monocyte apoptosis by the protein kinase Cdelta-dependent phosphorylation of caspase-3.** *Journal of Biological Chemistry* 2005, **280:**17371-17379.
2. Ceolotto G, Gallo A, Miola M, Sartori M, Trevisan R, Del Prato S, Semplicini A, Avogaro A: **Protein kinase C activity is acutely regulated by plasma glucose concentration in human monocytes in vivo.** *Diabetes* 1999, **48:**1316-1322.
3. Grinnell F: **Fibronectin and wound healing.** *Journal of Cellular Biochemistry* 1984, **26:**107-116.
4. Rawlings JS, Rosler KM, Harrison DA: **The JAK/STAT signaling pathway.** *Journal of Cell Science* 2004, **117:**1281-1283.
5. Gordon S: **Pattern recognition receptors: doubling up for the innate immune response.** *Cell* 2002, **111:**927-930.
6. Matikainen S, Hurme M: **Comparison of retinoic acid and phorbol myristate acetate as inducers of monocytic differentiation.** *International Journal of Cancer* 1994, **57:**98-103.
7. He H, Hua X, Yan J: **Epigenetic regulations in hematopoietic Hox code.** *Oncogene* 2011, **30:**379-388.

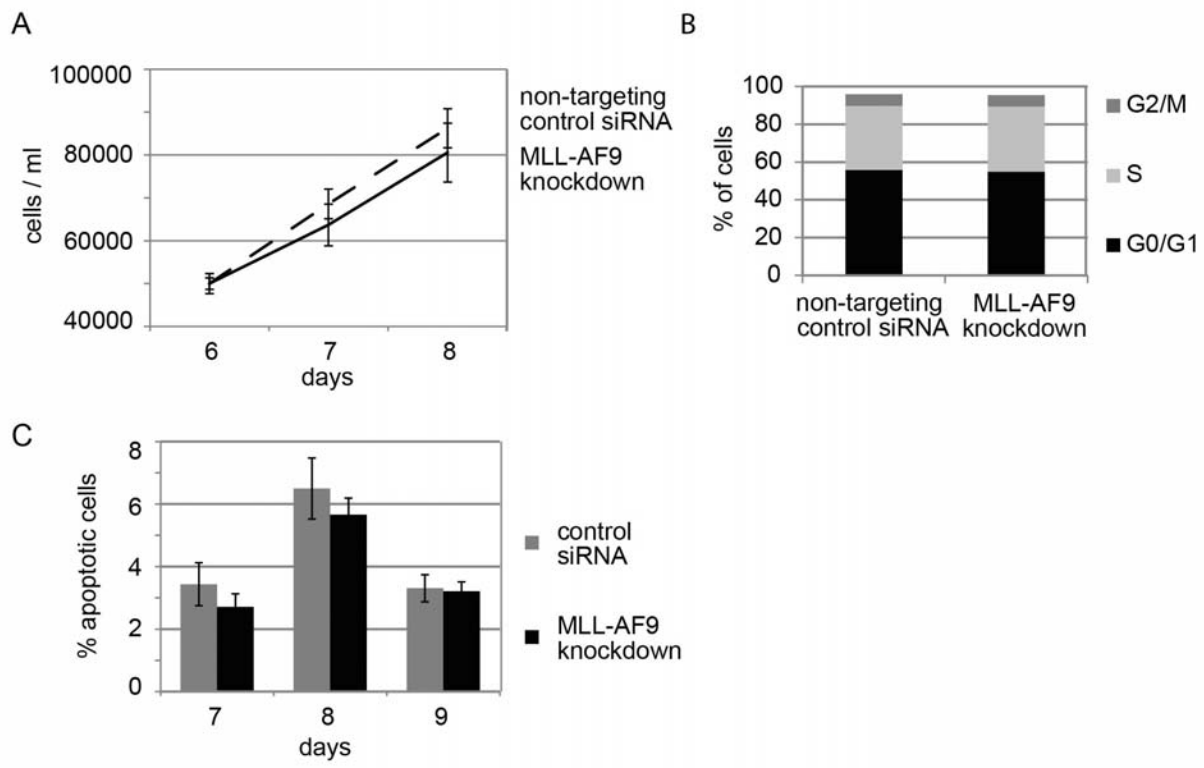

Figure S2: Cell characteristics of MLL-AF9 depleted and control THP1 cells. (A) Proliferation over five independent experiments. Bars indicate standard error. (B) Cell cycle distribution over three independent experiments. (C) Apoptosis rate in one experiment performed in triplicates is shown. Bars indicate standard error.

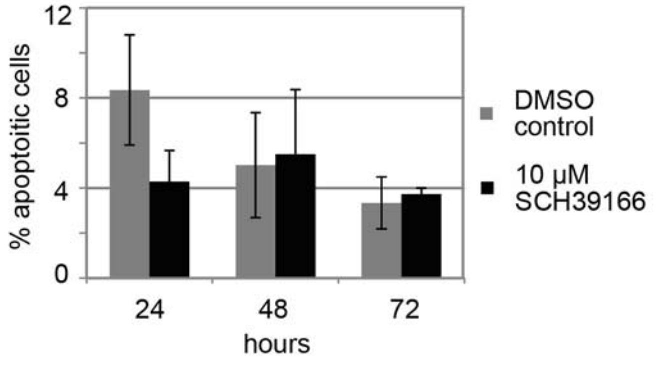

Figure S3: Apoptosis rate in THP1 cells treated with 10 µM SCH39166 compared to DMSO control. Columns indicate mean over three independent experiments. Bars indicate standard error of the mean.

Table S2: Biological roles and rating of 70 selected genes differentially expressed after MLL-AF9 knockdown. Genes were rated concerning the mediation of leukemogenic effects. Rating terms (based on biological role, link to leukemia and differential expression) are listed in table S4. * Indicates those candidates rated as likely mediators of MLL-AF9 leukemogenic effects which encode proteins of conventionally druggable classes (receptors, enzymes and transporter). log2FC ≥ ±1: strongest differentially expressed transcripts, FunDO: associated to leukemia via functional disease ontology, GOA: top differentially expressed genes within higher-order terms of functional gene ontology annotation, concordance with patient studies (references given).

**Gene** Rating category	**log_2 FC**	**Official full name** Biological role	**Selection criteria**
AHR* ++	0.44	**Aryl hydrocarbon receptor** AHR is a nuclear receptor. AHR is upregulated by AML associated fusion gene AML1-ETO [1]. Overexpression of AhR has been shown to promote retinoic acid–induced differentiation of myeloblastic leukemia cells. Within an ubiquitin ligase complex, AHR is important for estrogen receptor degradation. [2] AHR knockout mice display CML [3].	FunDO
ALOX5 -	0.76	**Arachidonate 5-lipoxygenase** Enzyme in the synthesis of leukotrienes (mediators of inflammatory and allergic conditions) from arachidonic acid. ALOX5 has functions in oxidative stress, inflammation and cancer and supports CML stem cell maintenance. [4]	GOA: calcium associated
ATP2B2* ++	-0.69	**ATPase, Ca++ transporting, plasma membrane 2** A plasma membrane pump which is activated by calmodulin and whose rat homolog (97.9% identical to human) was suggested to maintain low free cytosolic Ca^{2+} concentration [5]. ATP2B2 is down-regulated in PMA differentiated U937 monocytes [6]. Overexpression of ATP2B2 in breast cancer cells lowers intracellular calcium and protects from apoptosis [7]. Estradiol and estrogen decreases ATP2B2 expression and activity [8].	GOA: calcium associated
B2M +	-0.47	**Beta-2-microglobulin** A serum protein found in association with the major histocompatibility complex (MHC) class I heavy chain. B2M levels are predictive of the risk of a myelodysplastic syndrome evolving into an AML [9] and could detect a relapse of B-CLL and multiple myeloma [10].	FunDO
CALR ++	-0.51	**Calreticulin** CALR acts as a major Ca^{2+}-binding (storage) protein in the lumen of the endoplasmic reticulum [11]. It can also act as an important modulator of the regulation of gene transcription by nuclear hormone receptors [12]. In this context, CALR can inhibit the transcriptional activities of retinoic acid receptor and was shown to inhibit retinoic acid-induced differentiation in neuronal cells [12]. CALR inhibits the translation of CEBPA, which is a key myeloid transcription factor and a frequently disrupted in AML [13]. CALR can increase DNA synthesis and counteract irreversible growth arrest via translational inhibition of p21 (CDKN1A) [14].	FunDO
CCL2 ++	1.61	**Chemokine (C-C motif) ligand 2** CCL2 binds to chemokine (C-C motif) receptor 2 and 4 (CCR2 and CCR4), the former of which is also up-regulated in a MLL-AF9 knockdown specific manner in our data set. CCL2 displays chemotactic activity for monocytes and basophils. It is up-regulated in PMA differentiated U937 monocytes [6]. AML blasts have been described to produce different amounts of CCL2 which influences the number of monocytes migrating towards AML blasts [15]. Plasma levels of CCL2 are decreased in patients with AML of the subtype M4 and M5 [16]. Thus, low levels of CCL2 might lead to reduced immunosurveillance of M4 and M5 blasts. In this context, CCL2 has been proposed for adoptive immunotherapy [15].	FunDO, log_2FC > ±1, GOA: proliferation and apoptosis; mono/mΦ-differentiation; calcium associated

**Gene** Rating category	**log_2 FC**	**Official full name** Biological role	**Selection criteria**
CD14 ○	1.05	**CD14 molecule** A differentiation marker for maturating monocytes [17-19] and a pattern recognition receptor.	$Log_2FC > \pm 1$, GOA: proliferation and apoptosis; mono/mΦ-differentiation
CD209 ○	0.73	**CD209 molecule** A type II C-type lectin and bona fide marker for wound-healing and regulatory alternative (M2) macrophages which might impair anti-tumor immunity via enhanced IL-10 production [20]. On the other hand, CD209 was found to mediate adhesion between dendritic cells (DCs) and ICAM-3 on resting T cells and to be essential for DC-induced T cell proliferation [21].	GOA: calcium associated
CEBPB ++	0.53	**CCAAT/enhancer binding protein (C/EBP), beta** CEBPB activates the promoter of PU.1, a suppressor for AML [22]. CEBPB leads to differentiation and reduced proliferation of CML cells [23].	Concordance with patient study [24].
CGNL1 ++	-0.74	**Cingulin-like 1** In epithelial cells, CGNL1 protein was shown to inactivate RhoA and activate Rac1 activity [25]. In hematopoietic stem and progenitor cell, these proteins mediate signals required for self-renewal and are involved in proliferation, homing and interaction with the bone marrow niche [26].	GOA: other
CIITA ++	0.83	**Class II, major histocompatibility complex, transactivator** Transcriptional coactivator and master controller of MHC class II expression [27]. Many leukemias of the myeloid lineage are negative for MHC II which was proposed to reduce immunosurveillance via cytotoxic T cells [28]. Activity of CIITA is regulated via phosphorylation and can be induced by treatment with the differentiating agent PMA [29].	FunDO, GOA: regulation of transcription
CTSG ○	-0.43	**Cathepsin G** A serine protease with microbicidal and proinflammatory activity which is strongly expressed in neutrophils. CTSG degrades engulfed cell debris and enhances hematopoietic progenitor cell mobilization [30].	FunDO
DACH1 ++	-0.54	**Dachshund homolog 1 (Drosophila)** DACH1 is lost in poor prognosis invasive breast cancer and inhibits migration and invasion via IL8 repression [31]. In concordance with this, IL8 is up-regulated in a MLL-AF9 dependent manner in our study.	Concordance with patient studies [32-35].
DEFA3 ○	1.36	**defensin, alpha 3, neutrophil-specific** Antimicrobial protein involved in killing of phagocytosed bacteria via a non-oxidative mechanism [36]. In this setting possibly an indicator of differentiation.	$Log_2FC > \pm 1$, GOA: mono/mΦ-differentiation
DEXI ++	-0.56	**Dexi homolog (mouse)** A dexamethasone-induced transcript suspected to encode a membrane protein, that dimerizes either with itself or another protein [37]. Fusions between DEXI and CIITA have been found in 48% of AML cases previously defined as “normal karyotype” [38]. Its function is unknown.	Concordance with patient studies [32, 34].
DRD5* ++	-0.96	**Dopamine receptor D5** A G-protein coupled receptor which stimulates cAMP accumulation [39]. DRD5 is expressed on CD34+ hematopoietic progenitor cells and its expression is raised after G-CSF treatment. In these cells, dopamine receptor agonists activate Wnt signaling, induce migration and increase clonogenic capacity and repopulation [40].	GOA: proliferation and apoptosis; mono/mΦ-differentiation; calcium associated
EGR2 ++	0.81	**Early growth response 2** A transcription factor which inhibits cancer cell proliferation, activates macrophage-specific genes [41] and is up-regulated in PMA differentiated U937 monocytes [6]. EGR1-3 binding motifs were found activated and being within the core motifs explaining the expressional changes after PMA differentiation in THP1 cells [42]. Its mouse homolog is under-expressed in hematopoietic stem and progenitor cells after Mll-AF9 knock-in [43].	GOA: early development; regulation of transcription

**Gene** Rating category	**log_2 FC**	**Official full name** Biological role	**Selection criteria**
EMP1 ++	0.74	**Epithelial membrane protein 1** EMP1 is down-regulated in oral squamous cell carcinoma and a putative tumor suppressor [44]. EMP1 is down-regulated in human leukemic stem cells (LSC) as compared to normal HSC as well as by survivin, an oncogene overexpressed in AML and thus might be a candidate for selective anti-LSC therapy in AML [45].	GOA: other
FOS ++	0.88	**FBJ murine osteosarcoma viral oncogene homolog** FOS and FOSB binding motifs were found activated and being within the core motifs explaining the expressional changes after PMA differentiation in THP1 cells [42]. FOS and FOSB are components of the dimeric transcription factor AP-1, which mediates subunit and context dependent gene regulation in response to cytokines, growth factors, stress signals, oncogenic stimuli, bacterial and viral infections. AP-1 exerts effects on proliferation, differentiation and apoptosis [46].	GOA: proliferation and apoptosis; regulation of transcription; other; concordance with patient study [24].
FOSB ++	1.05	**FBJ murine osteosarcoma viral oncogene homolog B** see FOS.	$Log_2FC > \pm1$, concordance with patient study [24].
GPNMB ++	1.24	**Glycoprotein (transmembrane) nmb** A type I transmembrane glycoprotein that plays anti-proliferative, anti-tumorigenic and anti-invasive roles in prostrate carcinoma cells [47] and is involved in growth delay and reduction of metastatic potential in melanoma [48]. GPNMB is an immune inhibitory receptor up-regulated by BCR-ABL tyrosine kinase inhibitors. Its signaling pathway is essential for the inhibition of T-cell activation by antigen-presenting cells. [49]	$Log_2FC > \pm1$
HAPLN2 ○	-1.20	**Hyaluronan and proteoglycan link protein 2** Extracellular matrix protein. Previously assessed as brain specific [50], although leukocytes were not analyzed in the study.	$Log_2FC > \pm1$
HIPK2* ++	0.50	**Homeodomain interacting protein kinase 2** A tumor suppressor. HIPK2 phosphorylates a number of transcription factors and cofactors (e.g. PML, p53, Pax6, c-Myb, AML1) and may trigger (myeloid) differentiation, development and apoptosis [51, 52]. Mutations have been found in AML cases [53].	FunDO
HLA-B ++	-0.64	**Major histocompatibility complex, class I, B** MHC class I deficient tumor clones may escape T-cell immune responses, but might be more susceptible to NK-cell-mediated lysis [54].	FunDO
HLA-DMB ++	1.49	**Major histo-compatibility complex, class II, DM beta** Nonclassical MHC class II molecule. See HLA-DPA1	$Log_2FC > \pm1$, GOA: mono/mΦ-differentiation
HLA-DPA1 ++	1.60	**Major histo-compatibility complex, class II, DP alpha 1** Classical MHC class II molecule. MHC class II proteins are selectively expressed in a subset of antigen presenting cells [27]. Up-regulation of these transcripts has been associated with differentiation of monoblasts; THP1 cells were observed to have reduced MHC class II expression levels compared to mature monocytes [19]. Loss of MHC class II gene expression has been shown to be strikingly correlated with poor patient outcome and decreased immunosurveillance in large B-cell lymphoma patients [55].	$Log_2FC > \pm1$, GOA: mono/mΦ-differentiation
HLA-DPB1 ++	1.54	**Major histo-compatibility complex, class II, DP beta 1** Classical MHC class II molecule. See HLA-DPA1	FunDO, l $og_2FC > \pm1$, GOA: mono/mΦ-differentiation

**Gene** Rating category	**log_2 FC**	**Official full name** Biological role	**Selection criteria**
HLA-DQA1 ++	0.96	**Major histocompatibility complex, class II, DQ alpha 1** Classical MHC class II molecule. See HLA-DPA1	GOA: mono/mΦ-differentiation
HLA-DQB1 ++	1.19	**Major histo-compatibility complex, class II, DQ beta 1** Classical MHC class II molecule. See HLA-DPA1	$Log_2FC > \pm 1$, GOA: mono/mΦ-differentiation
HLA-DRB4 ++	0.45	**Major histocompatibility complex, class II, DR beta 4** Classical MHC class II molecule. See HLA-DPA1	Concordance with patient study [56].
HMBOX1 ○	-0.95	**Homeobox containing 1** A transcription factor / transcriptional repressor [57]. Key factor in differentiation of bone marrow stromal cells into vascular endothelial cells [58].	GOA: mono/mΦ-differentiation, early development; regulation of transcription
HOXA11 ++	-0.50	**Homeobox A11** HOXA11 (together with HOXA4, A7, A10 and MEIS1) is down-regulated after PMA induced differentiation in THP1 cells [59]. It is also down-regulated in PMA differentiated U937 monocytes [6]. HOXA11 has been found to be translocated to NUP98 in chronic myeloid leukemia [60].	FunDO
HOXA7 +	-0.41	**Homeobox A7** A leukemogenic factor which is, like other homeobox genes, frequently up-regulated in MLL aberrations and leukemia [61, 62]. Its mouse homolog is overexpressed in hematopoietic stem and progenitor cells after Mll-AF9 knock-in [43]. HOXA7 is down-regulated during differentiation of THP1 via PMA [59].	FunDO, concordance with patient study [33].
HOXA9 ++	-0.57	**Homeobox A9** A well-studied homeobox transcription factor whose transcription level is raised by MLL-AF9 through direct interaction between MLL-AF9 protein complex and HOXA9 promoter [63, 64]. Its mouse homolog is overexpressed in hematopoietic stem and progenitor cells after Mll-AF9 knockin [43]. Concerning hematopoiesis, HOXA9 knockout in mice has little or no effect on earlier progenitors but decreases the number of committed progenitors [65]. Overexpression of Hoxa9 alone does not lead to leukemic transformation of primary bone marrow cells in mice but does in conjunction with Meis1 overexpression [66]. Like BMI1, HOXA9 is essential for the self-renewal capacity of LCS transformed by MLL-AF9 [67].	Concordance with patient studies [33, 34].
HOXC4 ++	-0.76	**Homeobox C4** Hoxc4 supports proliferation and/or self-renewal of murine hematopoietic stem cells, myeloid and erythroid progenitors and may contribute to stem cell character of mesenchymal stem cells [68]. In vitro overexpression induces expansion of committed as well as very early human hematopoietic progenitors [69].	GOA: early development
HPX-2 ○	0.75	**Homeobox HPX-2** Homeobox containing gene, cloned from human CD34+ hematopoietic cells [70]. Biological role unknown.	GOA: early development
IFI30 ○	0.91	**Interferon, gamma-inducible protein 30** A lysosomal thiol reductase constitutively expressed in antigen-presenting cells with a putative role in MHC class II-restricted antigen processing. [71]	GOA: mono/mΦ-differentiation
IKZF2 ++	-0.60	**IKAROS family zinc finger 2 (Helios)** A transcription factor. A short isoform of IKZF2 is overexpressed in patients with adult T-cell leukemia / lymphoma and a role in leukemogenesis has been suggested [72].	FunDO

**Gene** Rating category	**log_2 FC**	**Official full name** Biological role	**Selection criteria**
IL8 ++	0.65	**Interleukin 8** A multifunctional chemokine, chemoattractant for neutrophils in inflammation, can influence basophils and T cell function and may act as an angiogenic, proliferative, autocrine and metastatic factor. IL8 is preferentially released by myeloid blasts showing monocytic differentiation (AML M4, M5). [73] Thus, up-regulation of the transcript might be associated with differentiation of monoblasts. DACH1 - which is down-regulated in our study - binds to the IL8 promoter and repressed it through AP1- and NFKB-binding sites. [31]	FunDO, concordance with patient study [24].
ITGAL ++	0.57	**Integrin, alpha L / CD11A** Adhesion molecule, up-regulated upon differentiation with all-trans retinoic acid in AML patients [74]. In B cell CLL with 11q22-q23 deletion, ITGAL and ITGAX show reduced expression [75].	FunDO
ITGAX ++	0.62	**Integrin, alpha X / CD11C** Adhesion molecule. Expression correlates with increased white blood cell count in AML, independent of FAB subtype and is up-regulated in AML patients upon differentiation with all-trans retinoic acid [74]. In B cell CLL with 11q22-q23 deletion, ITGAL and ITGAX show reduced expression [75].	FunDO
LGALS2 ○	1.02	**Lectin, galactoside-binding, soluble, 2** A galectin. Galectins are a group of proteins which have been implicated in the modulation of cell–cell and cell–matrix interactions [76]. LGALS2 was previously neither reasonably detected in untreated nor in differentiated HL-60 myeloid cells [77]. However, it is up-regulated in PMA differentiated U937 monocytes [6].	$Log_2FC > \pm1$,
LHX9 ○	-1.34	**LIM homeobox protein 9** A transcription factor repressed by Hoxb1 and mediating neuronal differentiation [78].	$Log_2FC > \pm1$, GOA: mono/mΦ-differentiation; early development; regulation of transcription
LOC 100129 722 ○	-1.32	**Hypothetical LOC100129722** Uncharacterized LOC100129722, non-coding RNA (NR_038389). Unknown function.	$Log_2FC > \pm1$
LOC 84989 ○	-1.06	**Hypothetical LOC84989** JMJD1C antisense RNA 1 (JMJD1C-AS1), non-coding RNA. Unknown function.	$Log_2FC > \pm1$
LPL ○	0.71	**Lipoprotein lipase** LPL plays a major role in the metabolism and transport of lipids and facilitates the uptake of lipoprotein particles, lipoprotein-associated lipids and lipophilic vitamins [79]. In THP-1 cells, LPL mRNA level is controlled via protein kinase C (PKC) and mobilization of intracellular Ca^{2+} and induced by PMA [80]. In CLL, high LPL expression may predict shorter overall survival [81].	FunDO
LRRC2 ○	-1.43	**Leucine rich repeat containing 2** This gene is located at chromosome 3p, a region commonly deleted in solid tumors [82]. Unknown function.	$Log_2FC > \pm1$
MEF2C ++	-0.60	**Myocyte enhancer factor 2C** A member of the MADS box transcription enhancer factor 2 (MEF2) family involved in myogenesis. Mef2c promotes myeloid progenitor proliferation and monopoiesis, is up-regulated in leukemic stem cells of MLL-associated leukemia and overexpressed in hematopoietic stem and progenitor cells after Mll-AF9 knockin but unable to induce leukemia when expressed alone [43, 83, 84].	Concordance with patient studies [34, 35].
MERTK ○	1.04	**C-mer proto-oncogene tyrosine kinase** Regulates macrophage cytokine secretion and clearance of apoptotic cells by macrophages and promotes macrophage survival in response to oxidative stress [85].	$Log_2FC > \pm1$, GOA: mono/mΦ-differentiation

**Gene** Rating category	**log_2 FC**	**Official full name** Biological role	**Selection criteria**
NAIP<br>-	0.60	**NLR family, apoptosis inhibitory protein**<br>Antiapoptotic protein, overexpressed in acute mixed lineage leukemia and was suggested to be involved in chemotherapy resistance [86].	FunDO
NPTX1<br>○	-1.00	**Neuronal pentraxin I**<br>NPTX1 is a pro-apoptotic protein in neurons [87].	$Log_2FC > \pm 1$, GOA: calcium associated
PARP8*<br>++	-0.63	**Poly (ADP-ribose) polymerase family, member 8**<br>PARP8 is phosphorylated upon DNA damage [88]. No published links to malignancies or leukemia.	Concordance with patient study [33].
PBX3<br>++	-0.51	**Pre-B-cell leukemia homeobox 3**<br>One isoform of PBX3 was suggested to have converse effects as compared to canonical PBX3 isoforms and is preferentially expressed in leukemic cells [89]. Within large protein complexes, PBX proteins regulate gene expression during developmental and/or differentiation processes. Overexpression of PBX3 might play a role in BCR/ABL-mediated myeloid differentiation and transformation. [90]	Concordance with patient studies [32-34].
PDCD5<br>-	-0.82	**Programmed cell death 5**<br>A proapoptotic protein, whose translocation to the nucleus has been described as a universal early event in apoptosis. PDCD5 transcript levels in AML and CML marrow cells are reduced as compared to normal donor marrow cells and are negatively correlated with BCR/ABL expression and function. Overexpression of PDCD5 enhances the chemosensitivity of K562 CML cells in vitro and in vivo. [91, 92]	FunDO
PDCD6IP<br>○	-0.46	**Programmed cell death 6 interacting protein**<br>Overexpression of PDCD6IP reestablishes contact inhibition of cell proliferation in Hela cells [93]. PDCD6IP overexpression promotes detachment-induced apoptosis, inhibits detachment of viable cells and reduces tumorigenicity in HeLa cells [94]. However, PDCD6IP overexpression also inhibits cell death induced by glucocorticoid in retinal pigment epithelial cells [95]. PDCD6IP is required for the completion of cytokinesis [96].	Concordance with patient study [32].
POT1<br>++	-0.94	**Protection of telomeres 1 homolog (S. pombe)**<br>POT1 protein binds single-stranded DNA from telomeres and is part of a large protein complex maintaining chromosome end stability [97]. Tumor cells can escape from replicative senescence by activating a telomere maintenance program [98].	GOA: proliferation and apoptosis
ROR2*<br>++	0.81	**Receptor tyrosine kinase-like orphan receptor 2**<br>A receptor / coreceptor for Wnt5a which mediates noncanonical Wnt signaling. Mutations within ROR2 are responsible for genetic skeletal disorders in humans. Oncogenic and tumor suppressive roles have been described for ROR2 in a wide array of malignancies. In leukemia, it is a putative tumor suppressor presumably via activating the Wnt5a/Ror2 noncanonical signaling pathway which inhibits Wnt canonical signaling. [99, 100]	GOA: early development
S100A12<br>++	0.84	**S100 calcium binding protein A12**<br>A calcium-binding protein, with multiple proinflammatory activities including chemotaxis for monocytes and neutrophils [101] which implicates a potential role in immunosurveillance. S100A12 is up-regulated in PMA differentiated U937 monocytes [6].	GOA: proliferation and apoptosis; calcium associated; concordance with patient study [24].
SGK1<br>-	1.10	**Serum/glucocorticoid regulated kinase 1**<br>SGK1 regulates channels, carriers, enzymes and transcription factors. It promotes survival, has antiapoptotic effects and may enhance the clonogenic capacity of cancer cells. [102]	$Log_2FC > \pm 1$

**Gene** Rating category	**log_2 FC**	**Official full name** Biological role	**Selection criteria**
SGMS1<br>-	0.75	**Sphingomyelin synthase 1**<br>SGMS1 synthesizes sphingomyelin and promotes cell proliferation [103]. Induction of cell death by the cytotoxic agent D609 in U937 monocytic leukemia cells might be achieved via inhibition of SGMS1 and decreased DAG levels [104].	GOA: other
SLC8A1<br>○	1.15	**Solute carrier family 8, member 1**<br>Although also expressed in other tissues (e.g. brain), SLC8A1 is the major sodium/calcium exchanger of the heart and is essential for heartbeats and survival of cardiac myocytes [105].	$Log_2FC > \pm1$, GOA: calcium associated
SNRPD2 P2<br>○	-1.13	**Small nuclear ribonucleoprotein D2 pseudogene 2 (LOC645339)**<br>A processed pseudogene with no evidence for protein coding function (Ensembl), although a 55 amino acid sequence is predicted. Unknown function. [106]	$Log_2FC > \pm1$
SOCS2<br>++	-1.07	**Suppressor of cytokine signaling 2**<br>SOCS2 is a negative regulator of cytokine receptor signaling via the Janus kinase pathway and suppresses the apoptotic effect of LIF [107]. SOCS2 may play a regulatory role in IGF1 receptor signaling [108], is induced by many cytokines and hormones (e.g. estrogen and GM-CSF) and up-regulated in advanced stages of CML but may have tumor suppressor roles in some solid tumors [109].<br>SOCS2 degrades SOCS3 presumably by mediating ubiquitinylation of SOCS3 [109]. In concordance with this observation, SOCS3 was up-regulated in our data set.	FunDO, $log_2FC > \pm1$, GOA: proliferation and apoptosis; mono/mΦ-differentiation; other; concordance with patient studies [24, 33-35].
SOCS3<br>○	0.53	**Suppressor of cytokine signaling 3**<br>SOCS3 blocks cytokine signaling by acting as kinase inhibitor of JAK proteins and as a binding competitor against STATs [109]. SOCS3 inhibits the therapeutic, CD33-induced block on proliferation in AML. A trend towards a higher reaction response and longer overall survival in patients with SOCS3 CpG hyper-methylation has been described [110]. On the other hand, SOCS3 can also inhibit non-receptor tyrosine kinases and has been reported to exhibit tumor suppressing activity, to inhibit proliferation and to enhance apoptosis presumably in a cell context dependent manner [111]. SOCS2 down-regulation in our data set may lead to the observed up-regulation of SOC3, because SOCS2 degrades SOCS3, likely by mediating its ubiquitinylation [109].	FunDO
SOX4<br>++	-0.84	**SRY (sex determining region Y)-box 4**<br>The transcription factor Sox4 directly up-regulated CREB expression and increases self-renewal and together with CREB also proliferation of mouse bone marrow progenitor cells. SOX4 is overexpressed in AML cell lines and AML patient samples. Sox4 cooperates with CREB in myeloid leukemia transformation. [112] Increased SOX4 expression due to proviral insertion was also identified as a cooperating event in myeloid leukemogenesis initiated by up-regulation of Evi1 or p15INK4b knockout in mice [113, 114].	GOA: proliferation and apoptosis; regulation of transcription
SP9<br>++	-1.19	**Sp9 transcription factor homolog (mouse)**<br>SP9 is involved in embryo limb outgrowth [115], in the formation of regeneration epithelium (like Hoxa-9 and Hoxa-13) and is a marker for dedifferentiation of keratinocytes in the axolotl [116].	$Log_2FC > \pm1$, GOA: mono/mΦ-differentiation
SYNPO2<br>-	-0.81	**Synaptopodin 2**<br>An actin associated protein described as tumor suppressor in urothelial carcinoma and prostate cancer that decreases invasiveness and cell proliferation [117].	GOA: other
TAS1R3*<br>++	-2.01	**Taste receptor, type 1, member 3**<br>A G-protein coupled receptor. A sweet taste receptor and glucose sensor which plays a role in regulation of glucose absorption in enterocytes and may be involved in energy supply [118]. TAS1R3 heterodimers also sense extracellular amino acids, activate MTORC1 and inhibit autophagy [119]. CEBPB, which was up-regulated in our study, activates TAS1R3 expression in a bile duct carcinoma cell line [120].	$Log_2FC > \pm1$

**Gene** Rating category	**log_2 FC**	**Official full name** Biological role	**Selection criteria**
VWA1 ○	-1.01	**Von Willebrand factor A domain containing 1** Encodes an extracellular matrix protein, which is expressed in skeletal and cardiac muscle, in cartilage tissue as well as in the apical ectodermal ridge of developing limb buds (like SP9) [121].	$Log_2FC > \pm1$, GOA: other
ZNF521 ++	-0.73	**Zinc finger protein 521** Highly expressed in the most immature hematopoietic cells and declines with differentiation. Enforced expression leads to proliferation and differentiation block, decreasing granulo-monocytic and erythroid differentiation. High ZNF521 expression has been associated with MLL rearranged AML and silencing ZNF521 in THP1 cells led to impaired growth and clonogenicity. [122] ZNF521 up-regulates HLA class I surface expression and enhances resistance of tumor cells to NK cell-mediated cytotoxicity [123].	FunDO, concordance with patient study [33].

References to Table S2:

1. Xu M, Li D, Lu Y, Chen GQ: **Leukemogenic AML1-ETO fusion protein increases carcinogen-DNA adduct formation with upregulated expression of cytochrome P450-1A1 gene.** *Exp Hematol* 2007, **35:**1249-1255.
2. Bunaciu RP, Yen A: **Activation of the aryl hydrocarbon receptor AhR promotes retinoic acid induced differentiation of myeloblastic leukemia cells by restricting expression of the stem cell transcription factor Oct4.** *Cancer Research* 2011, **71:**2371-2380.
3. Butler RW, Margaret; Gustafsson, Jan-Ake: **Chronic myeloid leukemia in the aryl hydrocarbon receptor knockout mouse.** In *Proceedings of the 104th Annual Meeting of the American Association for Cancer Research*, vol. Apr 6-10. Washington, DC.2013.
4. Naka K, Hoshii T, Hirao A: **Novel therapeutic approach to eradicate tyrosine kinase inhibitor resistant chronic myeloid leukemia stem cells.** *Cancer Science* 2010, **101:**1577-1581.
5. Elwess NL, Filoteo AG, Enyedi A, Penniston JT: **Plasma membrane Ca2+ pump isoforms 2a and 2b are unusually responsive to calmodulin and Ca2+.** *Journal of Biological Chemistry* 1997, **272:**17981-17986.
6. Wang J, Xiang G, Mitchelson K, Zhou Y: **Microarray profiling of monocytic differentiation reveals miRNA-mRNA intrinsic correlation.** *Journal of Cellular Biochemistry* 2011, **112:**2443-2453.
7. VanHouten J, Sullivan C, Bazinet C, Ryoo T, Camp R, Rimm DL, Chung G, Wysolmerski J: **PMCA2 regulates apoptosis during mammary gland involution and predicts outcome in breast cancer.** *Proceedings of the National Academy of Sciences of the United States of America* 2010, **107:**11405-11410.
8. El Beialy W, Galal N, Deyama Y, Yoshimura Y, Suzuki K, Tei K, Totsuka Y: **Effects of estrogen on PMCA 2 and 4 in human fibroblast-like synovial cells and mouse macrophage-like cells.** *Endocrine Journal* 2010, **57:**93-97.
9. Neumann F, Gattermann N, Barthelmes HU, Haas R, Germing U: **Levels of beta 2 microglobulin have a prognostic relevance for patients with myelodysplastic syndrome with regard to survival and the risk of transformation into acute myelogenous leukemia.** *Leukemia Research* 2009, **33:**232-236.

10. Melillo L, Musto P, Tomasi P, Cascavilla N, Bodenizza C, Ladogana S, Carotenuto M: **Serum beta 2-microglobulin in malignant lymphoproliferative disorders.** *Tumori* 1988, **74:**129-135.
11. Burns K, Duggan B, Atkinson EA, Famulski KS, Nemer M, Bleackley RC, Michalak M: **Modulation of gene expression by calreticulin binding to the glucocorticoid receptor.** *Nature* 1994, **367:**476-480.
12. Dedhar S, Rennie PS, Shago M, Hagesteijn CY, Yang H, Filmus J, Hawley RG, Bruchovsky N, Cheng H, Matusik RJ: **Inhibition of nuclear hormone receptor activity by calreticulin.** *Nature* 1994, **367:**480-483.
13. Schardt JA, Mueller BU, Pabst T: **Activation of the unfolded protein response in human acute myeloid leukemia.** *Methods in Enzymology* 2011, **489:**227-243.
14. Iakova P, Wang GL, Timchenko L, Michalak M, Pereira-Smith OM, Smith JR, Timchenko NA: **Competition of CUGBP1 and calreticulin for the regulation of p21 translation determines cell fate.** *EMBO Journal* 2004, **23:**406-417.
15. Legdeur MC, Broekhoven MG, Schuurhuis GJ, Beelen RH, Ossenkoppele GJ: **Monocyte-chemoattractant-protein-1-mediated migration of human monocytes towards blasts from patients with acute myeloid leukemia.** *Cancer Immunology, Immunotherapy* 2001, **50:**16-22.
16. Mazur G, Wrobel T, Butrym A, Kapelko-Slowik K, Poreba R, Kuliczkowski K: **Increased monocyte chemoattractant protein 1 (MCP-1/CCL-2) serum level in acute myeloid leukemia.** *Neoplasma* 2007, **54:**285-289.
17. Schwende H, Fitzke E, Ambs P, Dieter P: **Differences in the state of differentiation of THP-1 cells induced by phorbol ester and 1,25-dihydroxyvitamin D3.** *Journal of Leukocyte Biology* 1996, **59:**555-561.
18. Lutherborrow M, Bryant A, Jayaswal V, Agapiou D, Palma C, Yang YH, Ma DD: **Expression profiling of cytogenetically normal acute myeloid leukemia identifies microRNAs that target genes involved in monocytic differentiation.** *American Journal of Hematology* 2011, **86:**2-11.
19. Abrink M, Gobl AE, Huang R, Nilsson K, Hellman L: **Human cell lines U-937, THP-1 and Mono Mac 6 represent relatively immature cells of the monocyte-macrophage cell lineage.** *Leukemia* 1994, **8:**1579-1584.
20. Dominguez-Soto A, Sierra-Filardi E, Puig-Kroger A, Perez-Maceda B, Gomez-Aguado F, Corcuera MT, Sanchez-Mateos P, Corbi AL: **Dendritic cell-specific ICAM-3-grabbing nonintegrin expression on M2-polarized and tumor-associated macrophages is macrophage-CSF dependent and enhanced by tumor-derived IL-6 and IL-10.** *Journal of Immunology* 2011, **186:**2192-2200.
21. Geijtenbeek TB, Torensma R, van Vliet SJ, van Duijnhoven GC, Adema GJ, van Kooyk Y, Figdor CG: **Identification of DC-SIGN, a novel dendritic cell-specific ICAM-3 receptor that supports primary immune responses.** *Cell* 2000, **100:**575-585.
22. Mueller BU, Pabst T, Fos J, Petkovic V, Fey MF, Asou N, Buergi U, Tenen DG: **ATRA resolves the differentiation block in t(15;17) acute myeloid leukemia by restoring PU.1 expression.** *Blood* 2006, **107:**3330-3338.
23. Guerzoni C, Bardini M, Mariani SA, Ferrari-Amorotti G, Neviani P, Panno ML, Zhang Y, Martinez R, Perrotti D, Calabretta B: **Inducible activation of CEBPB, a gene negatively regulated by BCR/ABL, inhibits proliferation and promotes differentiation of BCR/ABL-expressing cells.** *Blood* 2006, **107:**4080-4089.
24. Zheng C, Li L, Haak M, Brors B, Frank O, Giehl M, Fabarius A, Schatz M, Weisser A, Lorentz C, Gretz N, Hehlmann R, Hochhaus A, Seifarth W: **Gene expression**

profiling of CD34+ cells identifies a molecular signature of chronic myeloid leukemia blast crisis. *Leukemia* 2006, **20:**1028-1034.
25. Guillemot L, Paschoud S, Jond L, Foglia A, Citi S: **Paracingulin Regulates the Activity of Rac1 and RhoA GTPases by Recruiting Tiam1 and GEF-H1 to Epithelial Junctions.** *Molecular Biology of the Cell* 2008, **19:**4442-4453.
26. Mulloy JC, Cancelas JA, Filippi MD, Kalfa TA, Guo F, Zheng Y: **Rho GTPases in hematopoiesis and hemopathies.** *Blood* 2010, **115:**936-947.
27. Ting JP, Trowsdale J: **Genetic control of MHC class II expression.** *Cell* 2002, **109 Suppl:**S21-S33.
28. Liu A, Takahashi M, Toba K, Zheng Z, Hashimoto S, Nikkuni K, Furukawa T, Koike T, Aizawa Y: **Regulation of the expression of MHC class I and II by class II transactivator (CIITA) in hematopoietic cells.** *Hematological Oncology* 1999, **17:**149-160.
29. Tosi G, Jabrane-Ferrat N, Peterlin BM: **Phosphorylation of CIITA directs its oligomerization, accumulation and increased activity on MHCII promoters.** *EMBO Journal* 2002, **21:**5467-5476.
30. Sun R, Iribarren P, Zhang N, Zhou Y, Gong W, Cho EH, Lockett S, Chertov O, Bednar F, Rogers TJ, Oppenheim JJ, Wang JM: **Identification of neutrophil granule protein cathepsin G as a novel chemotactic agonist for the G protein-coupled formyl peptide receptor.** *Journal of Immunology* 2004, **173:**428-436.
31. Wu K, Katiyar S, Li A, Liu M, Ju X, Popov VM, Jiao X, Lisanti MP, Casola A, Pestell RG: **Dachshund inhibits oncogene-induced breast cancer cellular migration and invasion through suppression of interleukin-8.** *Proceedings of the National Academy of Sciences of the United States of America* 2008, **105:**6924-6929.
32. Bullinger L, Dohner K, Bair E, Frohling S, Schlenk RF, Tibshirani R, Dohner H, Pollack JR: **Use of gene-expression profiling to identify prognostic subclasses in adult acute myeloid leukemia.** *New England Journal of Medicine* 2004, **350:**1605-1616.
33. Kohlmann A, Schoch C, Dugas M, Schnittger S, Hiddemann W, Kern W, Haferlach T: **New insights into MLL gene rearranged acute leukemias using gene expression profiling: shared pathways, lineage commitment, and partner genes.** *Leukemia* 2005, **19:**953-964.
34. Ross ME, Mahfouz R, Onciu M, Liu HC, Zhou X, Song G, Shurtleff SA, Pounds S, Cheng C, Ma J, Ribeiro RC, Rubnitz JE, Girtman K, Williams WK, Raimondi SC, Liang DC, Shih LY, Pui CH, Downing JR: **Gene expression profiling of pediatric acute myelogenous leukemia.** *Blood* 2004, **104:**3679-3687.
35. Valk PJ, Verhaak RG, Beijen MA, Erpelinck CA, Barjesteh van Waalwijk van D-K, Boer JM, Beverloo HB, Moorhouse MJ, van der Spek PJ, Lowenberg B, Delwel R: **Prognostically useful gene-expression profiles in acute myeloid leukemia.** *New England Journal of Medicine* 2004, **350:**1617-1628.
36. Harder J, Glaser R, Schroder JM: **Human antimicrobial proteins effectors of innate immunity.** *J EndotoxinRes* 2007, **13:**317-338.
37. Edgar AJ, Birks EJ, Yacoub MH, Polak JM: **Cloning of dexamethasone-induced transcript: a novel glucocorticoid-induced gene that is upregulated in emphysema.** *American Journal of Respiratory Cell and Molecular Biology* 2001, **25:**119-124.
38. Wen H, Li Y, Malek SN, Kim YC, Xu J, Chen P, Xiao F, Huang X, Zhou X, Xuan Z, Mankala S, Hou G, Rowley JD, Zhang MQ, Wang SM: **New fusion transcripts identified in normal karyotype acute myeloid leukemia.** *PLoS One* 2012, **7:**e51203.

39. Grandy DK, Zhang YA, Bouvier C, Zhou QY, Johnson RA, Allen L, Buck K, Bunzow JR, Salon J, Civelli O: **Multiple human D5 dopamine receptor genes: a functional receptor and two pseudogenes.** *Proceedings of the National Academy of Sciences of the United States of America* 1991, **88:**9175-9179.
40. Spiegel A, Shivtiel S, Kalinkovich A, Ludin A, Netzer N, Goichberg P, Azaria Y, Resnick I, Hardan I, Ben Hur H, Nagler A, Rubinstein M, Lapidot T: **Catecholaminergic neurotransmitters regulate migration and repopulation of immature human CD34+ cells through Wnt signaling.** *Nat Immunol* 2007, **8:**1123-1131.
41. Gabet Y, Baniwal SK, Leclerc N, Shi Y, Kohn-Gabet AE, Cogan J, Dixon A, Bachar M, Guo L, Turman JE, Jr., Frenkel B: **Krox20/EGR2 deficiency accelerates cell growth and differentiation in the monocytic lineage and decreases bone mass.** *Blood* 2010, **116:**3964-3971.
42. Suzuki H, Forrest AR, van Nimwegen E, Daub CO, Balwierz PJ, Irvine KM, Lassmann T, Ravasi T, Hasegawa Y, de Hoon MJ, Katayama S, Schroder K, Carninci P, Tomaru Y, Kanamori-Katayama M, Kubosaki A, Akalin A, Ando Y, Arner E, Asada M, Asahara H, Bailey T, Bajic VB, Bauer D, Beckhouse AG, Bertin N, Bjorkegren J, Brombacher F, Bulger E, Chalk AM, et al: **The transcriptional network that controls growth arrest and differentiation in a human myeloid leukemia cell line.** *Nature Genetics* 2009, **41:**553-562.
43. Chen W, Kumar AR, Hudson WA, Li Q, Wu B, Staggs RA, Lund EA, Sam TN, Kersey JH: **Malignant transformation initiated by Mll-AF9: gene dosage and critical target cells.** *Cancer Cell* 2008, **13:**432-440.
44. Zhang J, Cao W, Xu Q, Chen WT: **The expression of EMP1 is downregulated in oral squamous cell carcinoma and possibly associated with tumour metastasis.** *J Clin Pathol* 2011, **64:**25-29.
45. Fukuda S, Abe M, Onishi C, Taketani T, Purevsuren J, Yamaguchi S, Conway EM, Pelus LM: **Survivin selectively modulates genes deregulated in human leukemia stem cells.** *J Oncol* 2011, **2011:**946936.
46. Hess J, Angel P, Schorpp-Kistner M: **AP-1 subunits: quarrel and harmony among siblings.** *Journal of Cell Science* 2004, **117:**5965-5973.
47. Tsui KH, Chang YL, Feng TH, Chang PL, Juang HH: **Glycoprotein transmembrane nmb: an androgen-downregulated gene attenuates cell invasion and tumorigenesis in prostate carcinoma cells.** *Prostate* 2012, **72:**1431-1442.
48. Weterman MA, Ajubi N, van Dinter IM, Degen WG, van Muijen GN, Ruitter DJ, Bloemers HP: **nmb, a novel gene, is expressed in low-metastatic human melanoma cell lines and xenografts.** *International Journal of Cancer* 1995, **60:**73-81.
49. Schwarzbich MA, Gutknecht M, Salih J, Salih HR, Brossart P, Rittig SM, Grunebach F: **The immune inhibitory receptor osteoactivin is upregulated in monocyte-derived dendritic cells by BCR-ABL tyrosine kinase inhibitors.** *Cancer Immunol Immunother* 2012, **61:**193-202.
50. Spicer AP, Joo A, Bowling RA, Jr.: **A hyaluronan binding link protein gene family whose members are physically linked adjacent to chondroitin sulfate proteoglycan core protein genes: the missing links.** *Journal of Biological Chemistry* 2003, **278:**21083-21091.
51. Calzado MA, Renner F, Roscic A, Schmitz ML: **HIPK2: a versatile switchboard regulating the transcription machinery and cell death.** *Cell Cycle* 2007, **6:**139-143.
52. Gresko E, Ritterhoff S, Sevilla-Perez J, Roscic A, Frobius K, Kotevic I, Vichalkovski A, Hess D, Hemmings BA, Schmitz ML: **PML tumor suppressor is regulated by**

HIPK2-mediated phosphorylation in response to DNA damage. *Oncogene* 2009, **28:**698-708.
53. Li XL, Arai Y, Harada H, Shima Y, Yoshida H, Rokudai S, Aikawa Y, Kimura A, Kitabayashi I: **Mutations of the HIPK2 gene in acute myeloid leukemia and myelodysplastic syndrome impair AML1- and p53-mediated transcription. .** *Oncogene* 2007, **26:**7231-7239.
54. Algarra I, Garcia-Lora A, Cabrera T, Ruiz-Cabello F, Garrido F: **The selection of tumor variants with altered expression of classical and nonclassical MHC class I molecules: implications for tumor immune escape.** *Cancer Immunology, Immunotherapy* 2004, **53:**904-910.
55. Rimsza LM, Roberts RA, Miller TP, Unger JM, LeBlanc M, Braziel RM, Weisenberger DD, Chan WC, Muller-Hermelink HK, Jaffe ES, Gascoyne RD, Campo E, Fuchs DA, Spier CM, Fisher RI, Delabie J, Rosenwald A, Staudt LM, Grogan TM: **Loss of MHC class II gene and protein expression in diffuse large B-cell lymphoma is related to decreased tumor immunosurveillance and poor patient survival regardless of other prognostic factors: a follow-up study from the Leukemia and Lymphoma Molecular Profiling Project.** *Blood* 2004, **103:**4251-4258.
56. Rozovskaia T, Ravid-Amir O, Tillib S, Getz G, Feinstein E, Agrawal H, Nagler A, Rappaport EF, Issaeva I, Matsuo Y, Kees UR, Lapidot T, Lo CF, Foa R, Mazo A, Nakamura T, Croce CM, Cimino G, Domany E, Canaani E: **Expression profiles of acute lymphoblastic and myeloblastic leukemias with ALL-1 rearrangements.** *Proceedings of the National Academy of Sciences of the United States of America* 2003, **100:**7853-7858.
57. Zhang M, Chen S, Li Q, Ling Y, Zhang J, Yu L: **Characterization of a novel human HMBOX1 splicing variant lacking the homeodomain and with attenuated transcription repressor activity.** *Molecular Biology Reports* 2010, **37:**2767-2772.
58. Su L, Zhao H, Sun C, Zhao B, Zhao J, Zhang S, Su H, Miao J: **Role of Hmbox1 in endothelial differentiation of bone-marrow stromal cells by a small molecule.** *ACS Chemical Biology* 2010, **5:**1035-1043.
59. Martino V, Bianchera A, Reia L, Bussolati O, Fazzina R, Marino F, Montemurro L, Tonelli R, Pession A, Gazzola GC, Sala R: **Down-regulation of HOXA4 , HOXA7 , HOXA10 , HOXA11 and MEIS1 during monocyte-macrophage differentiation in THP-1 cells** *Molecular Medicine Reports* 2009, **2** 241-244.
60. Fujino T, Suzuki A, Ito Y, Ohyashiki K, Hatano Y, Miura I, Nakamura T: **Single-translocation and double-chimeric transcripts: detection of NUP98-HOXA9 in myeloid leukemias with HOXA11 or HOXA13 breaks of the chromosomal translocation t(7;11)(p15;p15).** *Blood* 2002, **99:**1428-1433.
61. Kawagoe H, Kawagoe R, Sano K: **Targeted down-regulation of MLL-AF9 with antisense oligodeoxyribonucleotide reduces the expression of the HOXA7 and -A10 genes and induces apoptosis in a human leukemia cell line, THP-1.** *Leukemia* 2001, **15:**1743-1749.
62. Afonja O, Smith JE, Jr., Cheng DM, Goldenberg AS, Amorosi E, Shimamoto T, Nakamura S, Ohyashiki K, Ohyashiki J, Toyama K, Takeshita K: **MEIS1 and HOXA7 genes in human acute myeloid leukemia.** *Leukemia Research* 2000, **24:**849-855.
63. Erfurth FE, Popovic R, Grembecka J, Cierpicki T, Theisler C, Xia ZB, Stuart T, Diaz MO, Bushweller JH, Zeleznik L: **MLL protects CpG clusters from methylation within the Hoxa9 gene, maintaining transcript expression.** *Proceedings of the National Academy of Sciences of the United States of America* 2008, **105:**7517-7522.

64. Cierpicki T, Risner LE, Grembecka J, Lukasik SM, Popovic R, Omonkowska M, Shultis DD, Zeleznik-Le NJ, Bushweller JH: **Structure of the MLL CXXC domain-DNA complex and its functional role in MLL-AF9 leukemia.** *Nature Structural & Molecular Biology* 2010, **17:**62-68.
65. Lawrence HJ, Helgason CD, Sauvageau G, Fong S, Izon DJ, Humphries RK, Largman C: **Mice bearing a targeted interruption of the homeobox gene HOXA9 have defects in myeloid, erythroid, and lymphoid hematopoiesis.** *Blood* 1997, **89:**1922-1930.
66. Kroon E, Krosl J, Thorsteinsdottir U, Baban S, Buchberg AM, Sauvageau G: **Hoxa9 transforms primary bone marrow cells through specific collaboration with Meis1a but not Pbx1b.** *EMBO Journal* 1998, **17:**3714-3725.
67. Smith LL, Yeung J, Zeisig BB, Popov N, Huijbers I, Barnes J, Wilson AJ, Taskesen E, Delwel R, Gil J, Van Lohuizen M, So CW: **Functional crosstalk between Bmi1 and MLL/Hoxa9 axis in establishment of normal hematopoietic and leukemic stem cells.** *Cell Stem Cell* 2011, **8:**649-662.
68. Phinney DG, Gray AJ, Hill K, Pandey A: **Murine mesenchymal and embryonic stem cells express a similar Hox gene profile.** *Biochemical and Biophysical Research Communications* 2005, **338:**1759-1765.
69. Daga A, Podesta M, Capra MC, Piaggio G, Frassoni F, Corte G: **The retroviral transduction of HOXC4 into human CD34(+) cells induces an in vitro expansion of clonogenic and early progenitors.** *Experimental Hematology* 2000, **28:**569-574.
70. Moretti P, Simmons P, Thomas P, Haylock D, Rathjen P, Vadas M, D'Andrea R: **Identification of homeobox genes expressed in human haemopoietic progenitor cells.** *Gene* 1994, **144:**213-219.
71. Arunachalam B, Phan UT, Geuze HJ, Cresswell P: **Enzymatic reduction of disulfide bonds in lysosomes: characterization of a gamma-interferon-inducible lysosomal thiol reductase (GILT).** *Proceedings of the National Academy of Sciences of the United States of America* 2000, **97:**745-750.
72. Tabayashi T, Ishimaru F, Takata M, Kataoka I, Nakase K, Kozuka T, Tanimoto M: **Characterization of the short isoform of Helios overexpressed in patients with T-cell malignancies.** *Cancer Science* 2007, **98:**182-188.
73. Vinante F, Rigo A, Tecchio C, Patuzzo C, Ricetti MM, Morosato L, Cassatella MA, Chilosi M, Pizzolo G: **Preferential release of high amounts of interleukin-8 by myeloid blasts showing monocytic differentiation.** *Haematologica* 1996, **81:**195-200.
74. Wu JJ, Cantor A, Moscinski LC: **beta2 Integrins are characteristically absent in acute promyelocytic leukemia and rapidly upregulated in vivo upon differentiation with all-trans retinoic acid.** *Leukemia Research* 2007, **31:**49-57.
75. Sembries S, Pahl H, Stilgenbauer S, Dohner H, Schriever F: **Reduced expression of adhesion molecules and cell signaling receptors by chronic lymphocytic leukemia cells with 11q deletion.** *Blood* 1999, **93:**624-631.
76. Wada J, Ota K, Kumar A, Wallner EI, Kanwar YS: **Developmental regulation, expression, and apoptotic potential of galectin-9, a beta-galactoside binding lectin.** *Journal of Clinical Investigation* 1997, **99:**2452-2461.
77. Abedin MJ, Kashio Y, Seki M, Nakamura K, Hirashima M: **Potential roles of galectins in myeloid differentiation into three different lineages.** *Journal of Leukocyte Biology* 2003, **73:**650-656.
78. Bami M, Episkopou V, Gavalas A, Gouti M: **Directed neural differentiation of mouse embryonic stem cells is a sensitive system for the identification of novel Hox gene effectors.** *PLoS One* 2011, **6:**e20197.

79. Wang H, Eckel RH: **Lipoprotein lipase: from gene to obesity.** *American Journal of PhysiologyEndocrinology and Metabolism* 2009, **297:**E271-E288.
80. Auwerx JH, Deeb S, Brunzell JD, Wolfbauer G, Chait A: **Lipoprotein lipase gene expression in THP-1 cells.** *Biochemistry* 1989, **28:**4563-4567.
81. Kaderi MA, Kanduri M, Buhl AM, Sevov M, Cahill N, Gunnarsson R, Jansson M, Ekstrom SK, Hjalgrim H, Jurlander J, Juliusson G, Mansouri L, Rosenquist R: **LPL is the strongest prognostic factor in a comparative analysis of RNA-based markers in early chronic lymphocytic leukemia.** *Haematologica* 2011, **96:**1153-1160.
82. Kiss H, Yang Y, Kiss C, Andersson K, Klein G, Imreh S, Dumanski JP: **The transcriptional map of the common eliminated region 1 (C3CER1) in 3p21.3.** *EurJ Hum Genet* 2002, **10:**52-61.
83. Krivtsov AV, Twomey D, Feng Z, Stubbs MC, Wang Y, Faber J, Levine JE, Wang J, Hahn WC, Gilliland DG, Golub TR, Armstrong SA: **Transformation from committed progenitor to leukaemia stem cell initiated by MLL-AF9.** *Nature* 2006, **442:**818-822.
84. Schuler A, Schwieger M, Engelmann A, Weber K, Horn S, Muller U, Arnold MA, Olson EN, Stocking C: **The MADS transcription factor Mef2c is a pivotal modulator of myeloid cell fate.** *Blood* 2008, **111:**4532-4541.
85. Anwar A, Keating AK, Joung D, Sather S, Kim GK, Sawczyn KK, Brandao L, Henson PM, Graham DK: **Mer tyrosine kinase (MerTK) promotes macrophage survival following exposure to oxidative stress.** *Journal of Leukocyte Biology* 2009, **86:**73-79.
86. Nakagawa Y, Hasegawa M, Kurata M, Yamamoto K, Abe S, Inoue M, Takemura T, Hirokawa K, Suzuki K, Kitagawa M: **Expression of IAP-family proteins in adult acute mixed lineage leukemia (AMLL).** *American Journal of Hematology* 2005, **78:**173-180.
87. Clayton KB, Podlesniy P, Figueiro-Silva J, Lopez-Domenech G, Benitez L, Enguita M, Abad MA, Soriano E, Trullas R: **NP1 regulates neuronal activity-dependent accumulation of BAX in mitochondria and mitochondrial dynamics.** *J Neurosci* 2012, **32:**1453-1466.
88. Matsuoka S, Ballif BA, Smogorzewska A, McDonald ER, III, Hurov KE, Luo J, Bakalarski CE, Zhao Z, Solimini N, Lerenthal Y, Shiloh Y, Gygi SP, Elledge SJ: **ATM and ATR substrate analysis reveals extensive protein networks responsive to DNA damage.** *Science* 2007, **316:**1160-1166.
89. Milech N, Kees UR, Watt PM: **Novel alternative PBX3 isoforms in leukemia cells with distinct interaction specificities.** *Genes, Chromosomes & Cancer* 2001, **32:**275-280.
90. Salesse S, Verfaillie CM: **BCR/ABL-mediated increased expression of multiple known and novel genes that may contribute to the pathogenesis of chronic myelogenous leukemia.** *Molecular Cancer Therapeutics* 2003, **2:**173-182.
91. Ruan GR, Qin YZ, Chen SS, Li JL, Ma X, Chang Y, Wang YZ, Fu JY, Liu YR: **Abnormal expression of the programmed cell death 5 gene in acute and chronic myeloid leukemia.** *Leukemia Research* 2006, **30:**1159-1165.
92. Ruan GR, Zhao HS, Chang Y, Li JL, Qin YZ, Liu YR, Chen SS, Huang XJ: **Adenovirus-mediated PDCD5 gene transfer sensitizes K562 cells to apoptosis induced by idarubicin in vitro and in vivo.** *Apoptosis* 2008, **13:**641-648.
93. Wu Y, Pan S, Che S, He G, Nelman-Gonzalez M, Weil MM, Kuang J: **Overexpression of Hp95 induces G1 phase arrest in confluent HeLa cells.** *Differentiation* 2001, **67:**139-153.

94. Wu Y, Pan S, Luo W, Lin SH, Kuang J: **Hp95 promotes anoikis and inhibits tumorigenicity of HeLa cells.** *Oncogene* 2002, **21:**6801-6808.
95. Valamanesh F, Torriglia A, Savoldelli M, Gandolphe C, Jeanny JC, BenEzra D, Behar-Cohen F: **Glucocorticoids induce retinal toxicity through mechanisms mainly associated with paraptosis.** *Mol Vis* 2007, **13:**1746-1757.
96. Carlton JG, Martin-Serrano J: **Parallels between cytokinesis and retroviral budding: a role for the ESCRT machinery.** *Science* 2007, **316:**1908-1912.
97. Barrientos KS, Kendellen MF, Freibaum BD, Armbruster BN, Etheridge KT, Counter CM: **Distinct functions of POT1 at telomeres.** *Molecular and Cellular Biology* 2008, **28:**5251-5264.
98. Finkel T, Serrano M, Blasco MA: **The common biology of cancer and ageing.** *Nature* 2007, **448:**767-774.
99. Yuan Y, Niu CC, Deng G, Li ZQ, Pan J, Zhao C, Yang ZL, Si WK: **The Wnt5a/Ror2 noncanonical signaling pathway inhibits canonical Wnt signaling in K562 cells.** *International Journal of Molecular Medicine* 2011, **27:**63-69.
100. Ford CE, Qian Ma SS, Quadir A, Ward RL: **The dual role of the novel Wnt receptor tyrosine kinase, ROR2, in human carcinogenesis.** *International Journal of Cancer* 2012, **133:**779-787.
101. Kishimoto K, Kaneko S, Ohmori K, Tamura T, Hasegawa K: **Olopatadine suppresses the migration of THP-1 monocytes induced by S100A12 protein.** *Mediators of Inflammation* 2006, **2006:**42726 42721-42725.
102. Lang F, Perrotti N, Stournaras C: **Colorectal carcinoma cells--regulation of survival and growth by SGK1.** *International Journal of Biochemistry & Cell Biology* 2010, **42:**1571-1575.
103. Shakor AB, Taniguchi M, Kitatani K, Hashimoto M, Asano S, Hayashi A, Nomura K, Bielawski J, Bielawska A, Watanabe K, Kobayashi T, Igarashi Y, Umehara H, Takeya H, Okazaki T: **Sphingomyelin synthase 1-generated sphingomyelin plays an important role in transferrin trafficking and cell proliferation.** *Journal of Biological Chemistry* 2011, **286:**36053-36062.
104. Meng A, Luberto C, Meier P, Bai A, Yang X, Hannun YA, Zhou D: **Sphingomyelin synthase as a potential target for D609-induced apoptosis in U937 human monocytic leukemia cells.** *Experimental Cell Research* 2004, **292:**385-392.
105. Wakimoto K, Kobayashi K, Kuro O, Yao A, Iwamoto T, Yanaka N, Kita S, Nishida A, Azuma S, Toyoda Y, Omori K, Imahie H, Oka T, Kudoh S, Kohmoto O, Yazaki Y, Shigekawa M, Imai Y, Nabeshima Y, Komuro I: **Targeted disruption of Na+/Ca2+ exchanger gene leads to cardiomyocyte apoptosis and defects in heartbeat.** *Journal of Biological Chemistry* 2000, **275:**36991-36998.
106. Thierry-Mieg D, Thierry-Mieg J: **AceView: a comprehensive cDNA-supported gene and transcripts annotation.** *Genome Biology* 2006, **7 Suppl 1:**S12-S14.
107. Minamoto S, Ikegame K, Ueno K, Narazaki M, Naka T, Yamamoto H, Matsumoto T, Saito H, Hosoe S, Kishimoto T: **Cloning and functional analysis of new members of STAT induced STAT inhibitor (SSI) family: SSI-2 and SSI-3.** *Biochemical and Biophysical Research Communications* 1997, **237:**79-83.
108. Dey BR, Spence SL, Nissley P, Furlanetto RW: **Interaction of human suppressor of cytokine signaling (SOCS)-2 with the insulin-like growth factor-I receptor.** *Journal of Biological Chemistry* 1998, **273:**24095-24101.
109. Rico-Bautista E, Flores-Morales A, Fernández-Pérez L: **Suppressor of cytokine signaling (SOCS) 2, a protein with multiple functions.** *Cytokine & Growth Factor Reviews* 2006, **17:**431-439.

110. Middeldorf I, Galm O, Osieka R, Jost E, Herman JG, Wilop S: **Sequence of administration and methylation of SOCS3 may govern response to gemtuzumab ozogamicin in combination with conventional chemotherapy in patients with refractory or relapsed acute myelogenous leukemia (AML).** *American Journal of Hematology* 2010, **85:**477-481.
111. Cooper JC, Shi M, Chueh FY, Venkitachalam S, Yu CL: **Enforced SOCS1 and SOCS3 expression attenuates Lck-mediated cellular transformation.** *Int J Oncol* 2010, **36:**1201-1208.
112. Sandoval S, Kraus C, Cho EC, Cho M, Bies J, Manara E, Accordi B, Landaw EM, Wolff L, Pigazzi M, Sakamoto KM: **Sox4 cooperates with CREB in myeloid transformation.** *Blood* 2012, **120:**155-165.
113. Boyd KE, Xiao YY, Fan K, Poholek A, Copeland NG, Jenkins NA, Perkins AS: **Sox4 cooperates with Evi1 in AKXD-23 myeloid tumors via transactivation of proviral LTR.** *Blood* 2006, **107:**733-741.
114. Bies J, Sramko M, Fares J, Rosu-Myles M, Zhang S, Koller R, Wolff L: **Myeloid-specific inactivation of p15Ink4b results in monocytosis and predisposition to myeloid leukemia.** *Blood* 2010, **116:**979-987.
115. Kawakami Y, Esteban CR, Matsui T, Rodriguez-Leon J, Kato S, Izpisua Belmonte JC: **Sp8 and Sp9, two closely related buttonhead-like transcription factors, regulate Fgf8 expression and limb outgrowth in vertebrate embryos.** *Development* 2004, **131:**4763-4774.
116. Satoh A, Graham GM, Bryant SV, Gardiner DM: **Neurotrophic regulation of epidermal dedifferentiation during wound healing and limb regeneration in the axolotl (Ambystoma mexicanum).** *Developmental Biology* 2008, **319:**321-335.
117. De Ganck A, De CV, Staes A, Gevaert K, Vandekerckhove J, Gettemans J: **Multiple isoforms of the tumor suppressor myopodin are simultaneously transcribed in cancer cells.** *Biochemical and Biophysical Research Communications* 2008, **370:**269-273.
118. Mace OJ, Lister N, Morgan E, Shepherd E, Affleck J, Helliwell P, Bronk JR, Kellett GL, Meredith D, Boyd R, Pieri M, Bailey PD, Pettcrew R, Foley D: **An energy supply network of nutrient absorption coordinated by calcium and T1R taste receptors in rat small intestine.** *The Journal of Physiology* 2009, **587:**195-210.
119. Wauson EM, Zaganjor E, Cobb MH: **Amino acid regulation of autophagy through the GPCR TAS1R1-TAS1R3.** *Autophagy* 2013, **9:**418-419.
120. Toyono T, Seta Y, Kataoka S, Toyoshima K: **CCAAT/Enhancer-binding protein beta regulates expression of human T1R3 taste receptor gene in the bile duct carcinoma cell line, HuCCT1.** *BiochimBiophys Acta* 2007, **1769:**641-648.
121. Allen JM, Brachvogel B, Farlie PG, Fitzgerald J, Bateman JF: **The extracellular matrix protein WARP is a novel component of a distinct subset of basement membranes.** *Matrix Biology* 2008, **27:**295-305.
122. Bond HM, Mesuraca M, Amodio N, Mega T, Agosti V, Fanello D, Pelaggi D, Bullinger L, Grieco M, Moore MAS, Venuta S, Morrone G: **Early hematopoietic zinc finger protein - zinc finger protein 521: A candidate regulator of diverse immature cells.** *International Journal of Biochemistry & Cell Biology* 2008, **40:**848-854.
123. La Rocca R, Fulciniti M, Lakshmikanth T, Mesuraca M, Ali TH, Mazzei V, Amodio N, Catalano L, Rotoli B, Ouerfelli O, Grieco M, Gulletta E, Bond HM, Morrone G, Ferrone S, Carbone E: **Early Hematopoietic Zinc Finger Protein Prevents Tumor Cell Recognition by Natural Killer Cells.** *Journal of Immunology* 2009, **182:**4529-4537.

Table S3: Primer used for reverse transcription quantitative PCR.

Gene name	Primer name	Orientation	Sequence 5'-3'	amplified product size	
AF9	AF9 (wt) F	forward	CAACGTTACCGCCATTTG	142	
AF9	AF9 (wt) R	reverse	GTCTGGGATGGTGTGAAG		
ARHGAP26	ARHGAP26 F	forward	CGAACTGGCCAAGGATTTCG	191	
ARHGAP26	ARHGAP26 R	reverse	GACGTTTCTCCTGCACGTAG		
CALR	CALR F	forward	GCCGAGCCTGCCGTCTACTTCA	201	
CALR	CALR R	reverse	AGGCTCGAAACTGGCCGACAG		
CEBPB	CEBPB F	forward	CAAACCAACCGCACATGCAG	264	
CEBPB	CEBPB R	reverse	AACAGCAACAAGCCCGTAGG		
CIITA	CIITA F	forward	GTGGGAGCCGAGAGCTTGGC	161	
CIITA	CIITA R	reverse	ATGGTGGGCGTCCACATCGC		
FOS	FOS F	forward	CGTACTCCAACCGCATCTGCA	636	
FOS	FOS R	reverse	CTCCGGTTGCGGCATTTGGC		
FUCA1	FUCA1 F	forward	GCTACGCCGACTTCGGACCG	183	
FUCA1	FUCA1 R	reverse	CCCGATGAGGCCCCACGTCT		
HOXA9	HOXA9 F	forward	CACCAGACGAACAGTGAGGA	252	[1]
HOXA9	HOXA9 R	reverse	TGGTCAGTAGGCCTTGAGGT		
MAFB	MAFB F	forward	GAAGCACCACCTGGAGAATG	120	
MAFB	MAFB R	reverse	GCGAGTTTCTCGCACTTGAC		
MLL1	MLL (wt) F	forward	GTCCAGAGCAGAGCAAAC	119	
MLL1	MLL (wt) R	reverse	CAAAGTGCCTGCATTCTCC		
MLL-AF9	MLL-AF9 F	forward	GCTCCCCGCCCAAGTATC	106	
MLL-AF9	MLL-AF9 R	reverse	GGGATGGTGTGAAGCTGGAG		
NOTCH2	NOTCH2 F	forward	ATATGCTCAGCCGGGATACC	214	
NOTCH2	NOTCH2 R	reverse	TGGCAGTGTCCTGGAATGTC		
RPL13A	RPL13A F	forward	CCTGGAGGAGAAGAGGAAAGAGA	126	[2]
RPL13A	RPL13A R	reverse	TTGAGGACCTCTGTGTATTTGTCAA		
SOCS2	SOCS2 F	forward	GCTCGGTCAGACAGGATGG	168	[3]
SOCS2	SOCS2 R	reverse	TCGATTCGAAGATTAGTTGGTCC		
SULF2	SULF2 F	forward	GTACAAGGCCAGCTATGTCC	257	
SULF2	SULF2 R	reverse	GGACTGTGTCGTTCTCTAGG		
TSAPAN14	TSAPAN14 F	forward	GGCTGGAGTTGTCTTCCTTG	109	
TSAPAN14	TSAPAN14 R	reverse	CAGCACCACAGGGTCGATTC		
UBC	UBC F	forward	ATTTGGGTCGCGGTTCTTG	133	[2]
UBC	UBC R	reverse	TGCCTTGACATTCTCGATGGT		
VASH1	VASH1 F	forward	ATGACTTCCGCAAGGAGCTG	159	
VASH1	VASH1 R	reverse	TTTCACTGCGGCTGTTCCTG		
VDR	VDR F	forward	GCCGCATCACCAAGGACAAC	121	
VDR	VDR R	reverse	ATCTCCCGCTTCCTCTGCAC		
ZNF521	ZNF521 F	forward	GGTGAAACTTGATATCAATGGCC	482	[4]
ZNF521	ZNF521 R	reverse	GGAGTTTGGCAGGAGAGTCA		

Unless indicated by citation, primers were designed utilizing Clone Manager Suite 7 (Sci-Ed Software, Cary, NC, USA).

References to Table S3:

1. Faber J, Krivtsov AV, Stubbs MC, Wright R, Davis TN, van den Heuvel-Eibrink M, Zwaan CM, Kung AL, Armstrong SA: **HOXA9 is required for survival in human MLL-rearranged acute leukemias.** *Blood* 2009, **113:**2375-2385.
2. Vandesompele J, De Preter K, Pattyn F, Poppe B, Van Roy N, De Paepe A, Speleman F: **Accurate normalization of real-time quantitative RT-PCR data by geometric averaging of multiple internal control genes.** *Genome Biology* 2002, **3:**research0034.0031-research0034.0012.
3. Zheng C, Li L, Haak M, Brors B, Frank O, Giehl M, Fabarius A, Schatz M, Weisser A, Lorentz C, Gretz N, Hehlmann R, Hochhaus A, Seifarth W: **Gene expression profiling of CD34+ cells identifies a molecular signature of chronic myeloid leukemia blast crisis.** *Leukemia* 2006, **20:**1028-1034.
4. La Rocca R, Fulciniti M, Lakshmikanth T, Mesuraca M, Ali TH, Mazzei V, Amodio N, Catalano L, Rotoli B, Ouerfelli O, Grieco M, Gulletta E, Bond HM, Morrone G, Ferrone S, Carbone E: **Early Hematopoietic Zinc Finger Protein Prevents Tumor Cell Recognition by Natural Killer Cells.** *Journal of Immunology* 2009, **182:**4529-4537.

Table S4: Rating strategy to prioritize candidate targets for mediation of MLL-AF9 leukemogenic effects. A total of 70 differentially expressed genes after MLL-AF9 knockdown was rated.

Category	Criteria
++	Candidates likely mediating MLL-AF9 leukemogenic effects fulfill at least two of the following three terms:<br>(1) Coherent regulation and described role in at least one of the following functions: functional role in differentiation of myeloid cells, proliferation, growth, apoptosis, survival, senescence, self-renewal, immunosurveillance, energy-supply, regeneration.<br>(2) Linked leukemia association via FunDO and / or agreement with published myeloid leukemia patient studies.<br>(3) Differential expression of the gene $\geq \pm 0.5$ log_2FC.
+	Genes whose regulation is coherent with their functional role in malignancies fulfill either term (1) or term (2).
○	Genes with a currently undefined role in malignancies (lack of relevant literature).
-	Genes whose regulation is incoherent with their functional role in malignancies or its association to leukemia.
